# The Salvinorin Analogue, Ethoxymethyl Ether Salvinorin B, Promotes Remyelination in Preclinical Models of Multiple Sclerosis

**DOI:** 10.3389/fneur.2021.782190

**Published:** 2021-12-20

**Authors:** Kelly F. Paton, Katharina Robichon, Nikki Templeton, Lisa Denny, Afnan Al Abadey, Dan Luo, Thomas E. Prisinzano, Anne C. La Flamme, Bronwyn M. Kivell

**Affiliations:** ^1^School of Biological Sciences, Victoria University of Wellington, Wellington, New Zealand; ^2^Centre for Biodiscovery, Victoria University of Wellington, Wellington, New Zealand; ^3^Department of Pharmaceutical Sciences, University of Kentucky, Lexington, KY, United States; ^4^Malaghan Institute of Medical Research, Wellington, New Zealand

**Keywords:** multiple sclerosis, kappa opioid receptor, experimental autoimmune encephalomyelitis, salvinorin A analog, remyelination, cuprizone-induced demyelination

## Abstract

Multiple sclerosis is a neurodegenerative disease associated with demyelination and neuroinflammation in the central nervous system. There is an urgent need to develop remyelinating therapies to better treat multiple sclerosis and other demyelinating diseases. The kappa opioid receptor (KOR) has been identified as a potential target for the development of remyelinating therapies; however, prototypical KOR agonists, such as U50,488 have side effects, which limit clinical use. In the current study, we investigated a Salvinorin A analog, ethoxymethyl ether Salvinorin B (EOM SalB) in two preclinical models of demyelination in C57BL/6J mice. We showed that in cellular assays EOM SalB was G-protein biased, an effect often correlated with fewer KOR-mediated side effects. In the experimental autoimmune encephalomyelitis model, we found that EOM SalB (0.1–0.3 mg/kg) effectively decreased disease severity in a KOR-dependent manner and led to a greater number of animals in recovery compared to U50,488 treatment. Furthermore, EOM SalB treatment decreased immune cell infiltration and increased myelin levels in the central nervous system. In the cuprizone-induced demyelination model, we showed that EOM SalB (0.3 mg/kg) administration led to an increase in the number of mature oligodendrocytes, the number of myelinated axons and the myelin thickness in the corpus callosum. Overall, EOM SalB was effective in two preclinical models of multiple sclerosis and demyelination, adding further evidence to show KOR agonists are a promising target for remyelinating therapies.

## Introduction

Multiple sclerosis (MS) is a devastating autoimmune disease characterized by the infiltration of autoreactive CD4 T cells in the central nervous system (CNS) leading to damage of the myelin sheaths surrounding axons, resulting in demyelination. MS affects approximately 2.8 million people worldwide (1) and can manifest in a range of different symptoms depending on the location of the lesions, including vision problems, cognitive impairments, and motor deficits that can ultimately lead to paralysis (2). MS can be broadly divided into three different subtypes: relapsing-remitting, primary progressive and secondary progressive. There is no cure for MS, with current disease-modifying treatments targeting the immune system to reduce damage to the myelin sheath formed by oligodendrocytes. However, the current treatments have limitations in preventing the progression of disability and are more successful at treating the relapsing-remitting forms of the disease (3). Thus, there is a current need to develop therapeutics that can induce remyelination, which could greatly benefit patients suffering from progressive forms of MS.

In 2016, activation of the kappa opioid receptor (KOR) was first shown to enhance oligodendrocyte progenitor cell (OPC) differentiation and remyelination (4, 5). Using the experimental autoimmune encephalomyelitis (EAE) preclinical mouse model of immune-mediated demyelination, KOR knockout mice exhibited a more severe disease progression, associated with enhanced demyelination and CNS infiltration (4). The prototypical KOR agonist, U50,488, significantly reduced EAE disease scores and enhanced remyelination in the cuprizone-induced demyelination model in mice (4). Validation that KOR is a remyelination target is supported by Tangherlini et al. (6) who showed that the novel quinoxaline class of KOR agonists were able to reduce disease severity in EAE. However, many KOR agonists, including U50,488, are not suitable for clinical development due to adverse side effects (7–11).

Salvinorin A is a neoclerodane diterpene with potent and selective KOR agonist actions (12). Salvinorin A analogs have been identified as having potential for the development as anti-addiction and anti-nociceptive therapeutics (13–16), however, they have not been assessed in models of MS. Ethoxymethyl ether Salvinorin B (EOM SalB) has been synthesized by altering the functional group at the carbon-2 position (17), which has led to increased binding affinity, potency and metabolic stability compared to Salvinorin A (18, 19). In addition, EOM SalB showed improved side effects. In rats, EOM SalB did not cause sedation in the spontaneous locomotor activity test or anxiety in the elevated plus-maze (19). The KOR is a G-protein coupled receptor, therefore, downstream signaling can occur via both the G-protein and β-arrestin pathways, with many of the negative side-effects, such as sedation and aversion, associated with the β-arrestin signaling pathways (20). The extremely G-protein biased KOR agonist, nalfurafine (21), significantly reduced EAE disease scores, increased remyelination and was more potent than U50,488 (22). Together these effects indicate that G-protein biased KOR agonists are highly effective at reducing EAE disease with reduced side effects and hold potential for the development of novel therapeutics to treat MS.

In the present study, we have assessed the G-protein bias of EOM SalB *in vitro* and used two complementary preclinical mouse models that recapitulate different aspects of MS to assess effects *in vivo*. The EAE model was used to assess immuno-modulatory effects in mice and the cuprizone-induced demyelination model was used to selectively kill the oligodendrocytes and model remyelination in the absence of peripheral immune cell infiltrations (23, 24).

## Materials and Methods

### Drug Preparation

EOM SalB was synthesized as previously described (18) and tested for purity (>99%) using high-performance liquid chromatography (HPLC). U50,488H and Salvinorin A were kindly provided by the National Institute on Drug Abuse Drug Supply Program.

### Cellular Assays

The cAMP Hunter™ CHO-K1 OPRK1 Gi Cell Line (catalog # 95-0088C2) and PathHunter® U2OS OPRK1 β-Arrestin Cell Line (catalog # 93-0234C3) were both purchased from Eurofins DiscoverX (Fremont, CA). The cAMP Hunter cell line was maintained in F-12 media supplemented with 10% fetal bovine serum (Life Technologies, Grand Island, NY), 1% penicillin/streptomycin/L-glutamine (Life Technologies), and 800 μg/mL Geneticin (Mirus Bio, Madison, WI). The PathHunter U2OS cell line was maintained in MEM media supplemented with 10% fetal bovine serum, 1% penicillin/streptomycin/L-glutamine, 500 μg/mL Geneticin, and 250 μg/ml Hygromycin B (Mirus Bio). All cells were grown at 37°C and 5% CO_2_ in a humidified incubator.

### Forskolin-Induced cAMP Accumulation

Following the previously described procedure (25), the cAMP HitHunter™ cells were seeded (10,000 cells/well) to 384-well tissue culture plates and incubated at 37°C overnight. The cells were treated with various doses of test compounds in the presence of forskolin for 30 min at 37°C followed by the detection using HitHunter cAMP assay for small molecules assay kit (Eurofins DiscoverX) according to the manufacturer's directions. BioTek Synergy H1 hybrid reader and Gen5 software (BioTek, Winooski, VT) were used to quantify the luminescence generated. Data were blank subtracted with vehicle control, normalized to forskolin controls, and analyzed with nonlinear regression using GraphPad Prism 8 software (GraphPad, La Jolla, CA).

### β-Arrestin2 Recruitment Assay

Following the previously described procedure (26) with modifications, the PathHunter™ cells were seeded (5,000 cells/well) into 384-well tissue culture plates and incubated at 37°C overnight. The cells were treated with various doses of test compounds for 30 min at 37°C followed by the detection using PathHunter detection kit (Eurofins DiscoverX) according to the manufacturer's directions. BioTek Synergy H1 hybrid reader and Gen5 software (BioTek, Winooski, VT) were used to quantify the luminescence generated. Data were blank subtracted with vehicle control, normalized to the reference compound U50,488, and analyzed with nonlinear regression using GraphPad Prism 8 software.

### Bias Calculation

The following formula, with U50,488 as the control ligand, was used to calculate the bias factor as previously described (16, 26, 27):


(1) $$\begin{array}{l} \log\left(bias\;factor\right)=\log{\left(\frac{E_{\max\left(test\right)}\times \;EC_{50\left(control\right)}}{EC_{50\left(test\right)}\times \;E_{\max\left(control\right)}}\right)}_{G-protein}\\ -\log{\left(\frac{E_{\max\left(test\right)}\times \;EC_{50\left(control\right)}}{EC_{50\left(test\right)}\times \;E_{\max\left(control\right)}}\right)}_{\beta -arrestin2\;} \end{array}$$

Using this formula, a bias factor of 1 is a balanced agonist, >1 is a G-protein biased agonist and <1 is a β-arrestin2 biased agonist, relative to U50,488.

### Animals

Female C57BL/6J mice (8–12 weeks; 18–27 g) were either acquired from the Malaghan Institute of Medical Research (Wellington, New Zealand) or were bred at the Victoria University of Wellington Animal Facility, New Zealand. The animals were group-housed (maximum 5 mice/cage) on a 12-hour light/dark cycle (lights on at 07:00) with stable temperature (19–21°C) and humidity (40–50%). Food and water were provided *ad libitum*. All procedures were carried out with the approval of the Victoria University of Wellington Animal Ethics Committee (approval numbers 25295, 24383 and 28154). All procedures were carried out in agreement with the New Zealand Animal Welfare Act, 1999.

EOM SalB and U50,488 were dissolved in a vehicle containing DMSO, Tween-80 (Sigma-Aldrich, St. Louis, MO) and 0.9% saline at a ratio of 1:1:8, respectively. The drugs were delivered at a volume of 5 μL/g of body weight via daily intraperitoneal (i.p.) injection. The KOR antagonist, nor-binaltorphimine (nor-BNI) was purchased from Tocris Bioscience and dissolved using a 0.9% saline vehicle.

### Experimental Autoimmune Encephalomyelitis (EAE) Model

Mice were immunized via subcutaneous (s.c.) injection in the rear flanks with myelin oligodendrocyte glycoprotein (MOG)_35−55_ peptide (50 μg/ mouse; Genescript, Piscataway, NJ) in complete Freund's adjuvant (Sigma-Aldrich) containing 500 μg/mouse heat-inactivated Mycobacterium tuberculosis H37Ra (Fort Richard, Auckland, New Zealand). In addition, mice were injected i.p. with pertussis toxin (200 ng/mouse; List Biochemicals, Campbell, CA) on days 0 and 2. Mice were weighed and scored daily as follows: 0, normal; 1, partial tail paralysis; 2, full tail paralysis; 3, paralysis in one hind limb; 4, paralysis in both hind limbs; and 5, moribund. Treatments were blinded throughout the experiment and initiated at disease onset (score ≥ 1). Mice were allocated consecutively to each treatment group upon disease onset to ensure the treatment regime remained even across groups. Drugs and vehicle control were administered daily by i.p. injection. Treatment with the selective KOR antagonist nor-BNI was performed weekly at 10 mg/kg i.p. with the first dose on the day of disease onset, followed by daily KOR treatments from 24 h post-nor-BNI treatment. Following CO_2_ euthanasia, spleens were isolated, and mice were perfused with phosphate-buffered saline (PBS, 140 mM NaCl, 2.68 mM KCl, 8.1 mM Na_2_HPO_4_, 1.47 mM KH_2_PO_4_, pH 7.4) followed by isolation of the brain tissue.

### Primary Cell Isolation Into Single-Cell Suspension

The brain tissue was mashed through a 70 μm cell strainer and centrifuged at 760 x g for 5 min followed by cell pellet resuspension in 37% Percoll™ gradient and centrifuged 30 min at 760 x g without brakes. Myelin layer was removed, supernatant discharged, and the pellet resuspended for flow cytometry. The spleen was mashed through a 70 μm cell strainer and centrifuged at 760 x g for 5 min, pellet was loosened and resuspended in Red Cell Lysis buffer for 2 min. Afterwards, cell pellets were resuspended and counted in preparation for flow cytometry.

### Analysis of Cytokines

Splenocytes were plated in complete T-cell medium in a round-bottomed 96-well plate (Corning, NY USA) and stimulated with medium, MOG_35−55_ peptide (27 μg/mL) or Concanavalin A (ConA) (1 μg/mL; Sigma-Aldrich). Cells were then incubated for 72 h at 37°C and 5% CO_2_. For intracellular cytokine analysis, splenocyte cultures were stimulated with phorbol 12-myristate 13- acetate (PMA; 50 ng/mL; Sigma-Aldrich) and ionomycin (500 ng/mL; Sigma-Aldrich) in the presence of GolgiStop/monensin (1 μg/10^6^ cells; BD Biosciences, NJ) for 4 h at 37°C and 5% CO_2_ before preparing for flow cytometry.

### Flow Cytometry

Cells were incubated with Fc Block (1 μg/10^6^ cells; 2.4G2; BD Biosciences) for 15 min. Extracellular staining was performed for 30 min on ice using the following antibodies: CD4-BV421 (RM4-5; BioLegend, San Diego, CA, USA), CD45-BV510 (30-F11; BioLegend), CD3-APC (17.A2, BioLegend), CD25-PE-Cy7 (PC61; BioLegend), CD8 PerCPCy5.5 (53-6.7; BioLegend), B220-APC-Cy7 (RA3-6B2; BD Biosciences), CD11b-PE-Cy7 (M1/70; BioLegend), Ly6C-PE (HK1.4; BioLegend), Gr1-APC-Cy7 (RB6-8C5; BioLegend), IA/IE-BV421 (M5/114.15.2; BioLegend), F4/80-FITC (BM8; BioLegend), CD11c-PerCP-Cy5.5 (N418; BioLegend).

After staining for extracellular proteins, cells were fixed in 4% paraformaldehyde (PFA, pH 7.4) and permeabilized using 0.1% saponin buffer containing 0.1% bovine serum albumin. For intracellular cytokine detection, interferon-γ (IFNγ)-BV421 (XMG 1.2, BioLegend), interleukin (IL)-10-PE (54902, BD Bioscience), and IL-17A-AF647 (TC11-18H10, BioLegend) antibodies were used.

Flow cytometry was performed on a BD FACS Canto II (BD Biosciences) and analyzed using FlowJo software version 10.1 (Treestar Inc., Ashland, OR, USA).

### Histological Techniques

Following CO_2_ euthanasia and perfusion with PBS, spinal cords were fixed in 4% paraformaldehyde overnight at 4°C. The cervical spinal cord was isolated and processed in a tissue processor (Leica TP1020, Wetzler, Germany) before paraffin embedding using a Leica embedding station (EG-1160). Transverse sections of 5–7 μm thickness were cut using a Leica RM 2235 microtome and mounted on Superfrost-plus slides (ThermoFisher) for histological staining.

Myelin was detected using luxol fast blue stain (LFB; Sigma-Aldrich). The protocol previously generated for Black gold II staining was modified (22) and is shown in Supplementary Figure 1. Infiltrating immune cells in the spinal cord were detected using hematoxylin and eosin (H&E) stain and is shown in Supplementary Figure 2. Using ImageJ software (version 1.52a, National Institutes of Health, Bethesda, MD, US), images of H&E-stained sections were converted to a red, green, blue (RGB) stack, and the red filter was selected. Using the selected region of interest, the threshold was set to include infiltrating cell nuclei. The white matter area was selected and the percentage area of infiltration was measured. Histological analysis was carried out on 2 sections per animal and these sections averaged to generate a mean value for each animal.

### Cuprizone-Induced Demyelination Model

Cuprizone (0.3%, Santa Cruz Biotechnology, TX, USA) was mixed into powdered food (Specialty Feeds, WA, Australia) and administered for 42 days at 5 g per mouse in a dish placed in the cage and replaced daily (28). Healthy control mice were given equivalent amounts of normal powdered food. After 42 days of cuprizone intoxication, all mice were given normal pelleted food. Drug treatment began on day 35, with treatments assigned for even distribution of weight loss across the groups. For the cuprizone model, EOM SalB was administered at 0.3 mg/kg and U50,488 at 1.6 mg/kg. Healthy control mice received vehicle injections. Mice were weighed daily with percentage change calculated using the baseline weight measured on day 0.

### Mechanical Sensitivity

An electronic von Frey anesthesiometer with the #7 Supertip filament (2390 series, IITC Life Science, CA, USA) was used to measure mechanical sensitivity. Mice were placed in a transparent chamber on top of a mesh stand and the filament was applied to the hind paw. The filament was advanced until the mouse displayed a withdrawal response, with each of the hind paws measured in triplicate. The experimenter was blinded to the treatment group during measurement collection.

### The Horizontal Bar Test

The triple horizontal bar test was carried out as previously described (29, 30). The three horizontal bars were 2, 4, and 6 mm in diameter and 38 cm in length and suspended 50 cm above the bench. The mice were placed on the middle of the bar, only grasping by their front paws and were timed for 30 s, with the time stopped if the mice traveled to the end of the bar or fell from the apparatus. A score was given based on the time the mouse remained on the apparatus: 1 = 0–5 s; 2 = 6–10 s; 3 = 11–20 s; 4 = 21–29 s; 5 = 30 s or traveled to the end of the bar. Testing began with the smallest, 2 mm, diameter bar. If a mouse scored a 5 it would advance to the next bar, however, if the mouse scored below 5, it would be repeated two more times and would not advance to the next bar. Scores were added across all the bars the mouse was tested on. The experimenter was blinded to the treatment group during measurement collection.

### Immunohistochemistry

Mice were deeply anesthetized with pentobarbital and transcardially perfused with 5% heparinized PBS followed by 4% PFA and the brain tissue was dissected and fixed overnight. The tissue was cryoprotected with 30% sucrose in PBS overnight, and cut using brain matrix at approximately bregma +2 to −2 mm before embedding in cryo-mountant and snap freezing in isopentane on a bed of dry ice. Coronal 20 μm sections were cut using a Leica CM3050 S cryostat microtome and free-floated onto Superfrost-plus slides (ThermoFisher).

Sections were washed with PBS and underwent antigen retrieval with EDTA buffer (1 mM EDTA, 0.05% Tween-20, pH 8) at 70°C for 15 min and incubated with 3 mg/mL glycine quench for 2 x 10 min. The slides were washed with PBST (PBS with 0.3% TrixonX-100) and blocked with 4% donkey serum in PBST for 2 h. The primary antibodies for SOX10 (1:500, R&D Systems, AF2864) and GST-pi (1:200, Enzo Life Sciences, ADI-MSA-102) were added to 4% donkey serum in PBST and incubated overnight at 4°C. The slides were washed with PBST and incubated with the secondary antibodies: donkey anti-goat Alexa Fluor 488 (1:500, ThermoFisher) and donkey anti-rabbit Alexa Fluor 555 (1:500, ThermoFisher) in 4% donkey serum in PBST for 3 h at room temperature. The slides were washed with PBS, DAPI solution (300 nM in PBS) added for 10 min, further washed with PBS and mounted with Prolong Gold mounting media (Invitrogen).

Slides were imaged on an Olympus FV3000 confocal microscope equipped with a 20x objective (Olympus, New Zealand). Each section was imaged at the midline of the corpus callosum. Three focal planes 4 μm apart were acquired and projected into a single image. The images were imported into ImageJ software to crop to the region of interest across all channels (612 x 138 μm). Three sections per mouse were analyzed with Cell Profiler software (version 3.1.9) to count the number of SOX10-positive and GST-pi-positive cells that were co-localized with DAPI-positive staining, using the Otsu thresholding method and the following modules: “ColourToGray”, “IdentifyPrimaryObjects”, “RelateObjects”, “FilterObjects” and “OverlayOutlines”.

### Transmission Electron Microscopy

Mice were deeply anesthetized with pentobarbital and transcardially perfused using a prewash of 5% heparin in PBS (pH 7.4) followed by the primary Karnovsky fixative (0.1 M sodium cacodylate buffer, 4% PFA, 0.25% glutaraldehyde, pH 7.4). The brain was removed and using a brain matrix, a 1 mm coronal brain slice, from approximately bregma 0 to −1 mm, was placed into a modified Karnovsky fixative (0.1 M sodium cacodylate buffer, 4% PFA, 2.5% glutaraldehyde, pH 7.4) and stored overnight at 4°C. The brain slice was dissected to isolate the midline of the corpus callosum under a dissecting microscope. The tissue underwent secondary fixation in 1% osmium tetroxide for 2 h, washed in distilled water, and a final tertiary fixation in 1% uranyl acetate for 2 h. The samples were dehydrated with ethanol, replaced with acetone and embedded in epoxy resin (Sigma-Aldrich), which was polymerized at 60°C for 48 h.

Semi-thin sections were cut on a Leica UC7 ultramicrotome and stained with toluidine blue to determine the correct area (corpus callosum). Transverse ultrathin sections (85 nm thickness) were cut with a diamond knife (Diatome, 45° angle) and stained using an LKB ultrastainer with lead citrate and uranyl acetate. Images were taken at 5,800x magnification on a Phillips CM100 TEM at the Otago Micro and Nanoscale Imaging unit located at the University of Otago, Dunedin, New Zealand.

The analysis of the transmission electron microscopy (TEM) images was carried out within a region of interest (9,736 x 8,095 nm) using ImageJ software. The number of myelinated and unmyelinated axons in the region of interest were counted manually. For the g-ratio assessment, the outer and inner axonal diameter lengths were measured for each axon within the region, and the g-ratios were calculated by dividing the inner axonal diameter by the outer axonal diameter. TEM analysis was carried out on 5 images per animal. The assessor was blinded from the treatment of each image.

### Statistical Analysis

GraphPad Prism (version 7.05, GraphPad Software, La Jolla, CA, USA) was used to determine statistical significance. Values are presented as the mean ± standard error of the mean (SEM). Comparisons between two groups were performed using a paired Student's t-test. For comparison of more than two groups, one-way or two-way analysis of variance (ANOVA) was used with the recommended multiple comparison tests as indicated in the figure legend and as recommended by GraphPad Prism. Comparisons were considered significant when *p* < 0.05.

## Results

### EOM SalB Is G-Protein Biased *in vitro*

**Figure 1:**
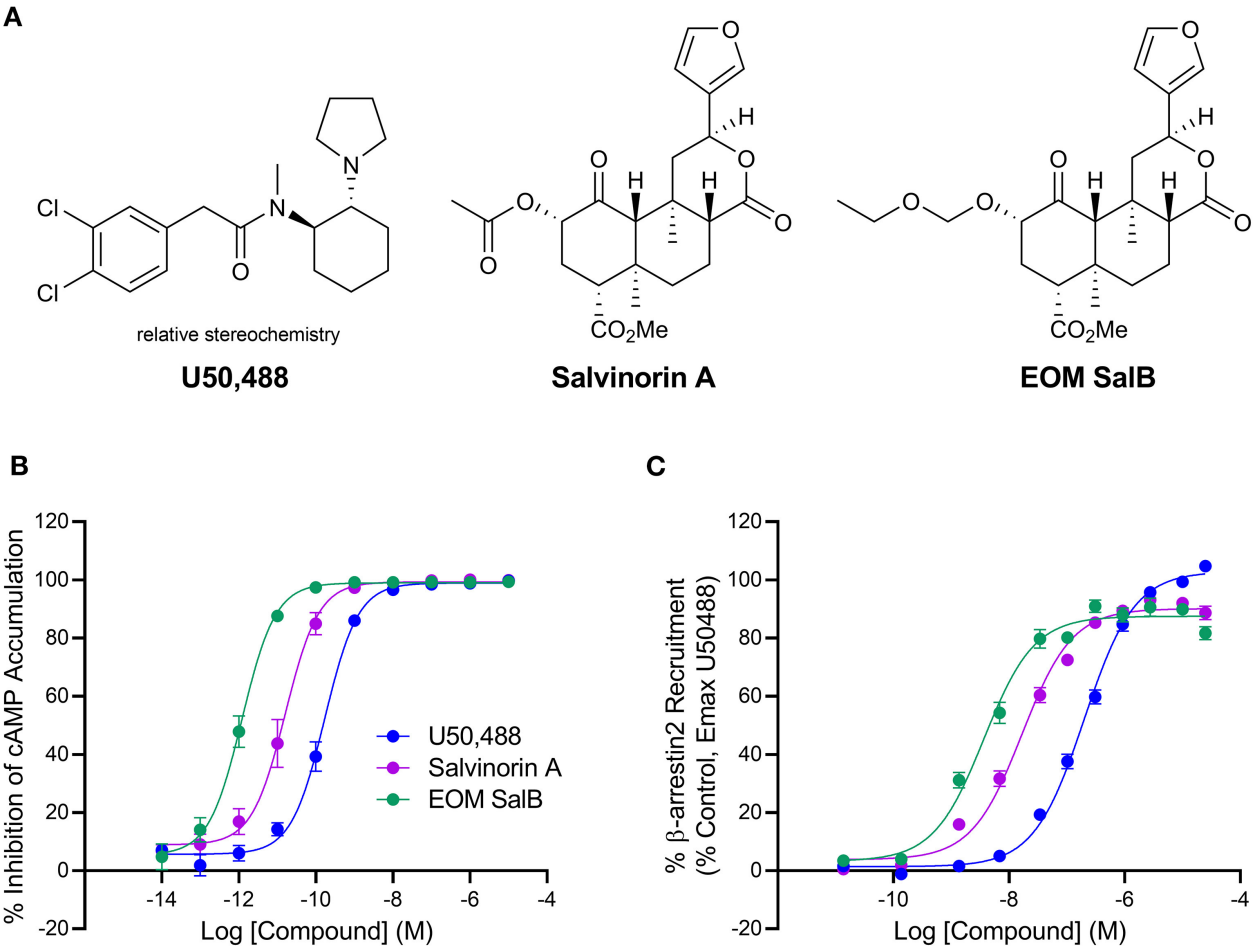
Chemical structures and *in vitro* activity. **(A)** Chemical structures of U50,488, Salvinorin A, and EOM SalB. **(B)** The KOR agonists were measured for inhibition of forskolin-induced cyclic adenosine monophosphate (cAMP) using the HitHunter™ assay. **(C)** The β-arrestin2 signaling pathway was assessed using the PathHunter™ assay of β-arrestin2 recruitment. Results from ≥3 experiments performed in triplicate. Values presented as mean ± SEM.

EOM SalB is an analog of Salvinorin A with alteration at the carbon-2 position, which is structurally distinct from the prototypical KOR agonist U50,488 (Figure 1A). The G-protein bias of EOM SalB was measured using the HitHunter assay measuring inhibition of forskolin-induced cAMP accumulation to assess the G-protein signaling, and compared to the PathHunter assay assessing the β-arrestin recruitment. Using U50,488 as the reference ligand, the results show that EOM SalB was more potent than the parent compound Salvinorin A and U50,488 in both assays (Figures 1B,C, Table 1). The bias calculation showed that EOM SalB was G-protein biased compared to U50,488 with a factor of 2.53, whereas Salvinorin A had a bias factor of 0.648 (Table 1).

**Table 1:** The *in vitro* activity of U50,488, Salvinorin A and EOM SalB.

	**Inhibition of forskolin-induced cAMP accumulation (HitHunter™)**		**β-arrestin2 recruitment (PathHunter™)**		
	**EC_50_ (nM)**	**E_max_ (%)**	**EC_50_ (nM)**	**E_max_ (%)**	**Bias Factor**
U50,488	0.181 ± 0.060	99.0 ± 0.8	231.8 ± 36.6	103.8 ± 0.5	1
Salvinorin A	0.0262 ± 0.0114	100.1 ± 0.3	18.8 ± 2.9	90.8 ± 2.0	0.648
EOM SalB	0.0015 ± 0.0008	99.2 ± 0.3	4.2 ± 1.2	87.7 ± 2.7	2.53

*Results from ≥3 experiments performed in triplicate and are calculated from the data presented in Figure 1. Values are mean ± SEM*.

### Therapeutic Treatment With EOM SalB Enabled Functional Recovery of EAE *via* KOR Activation

**Figure 2:**
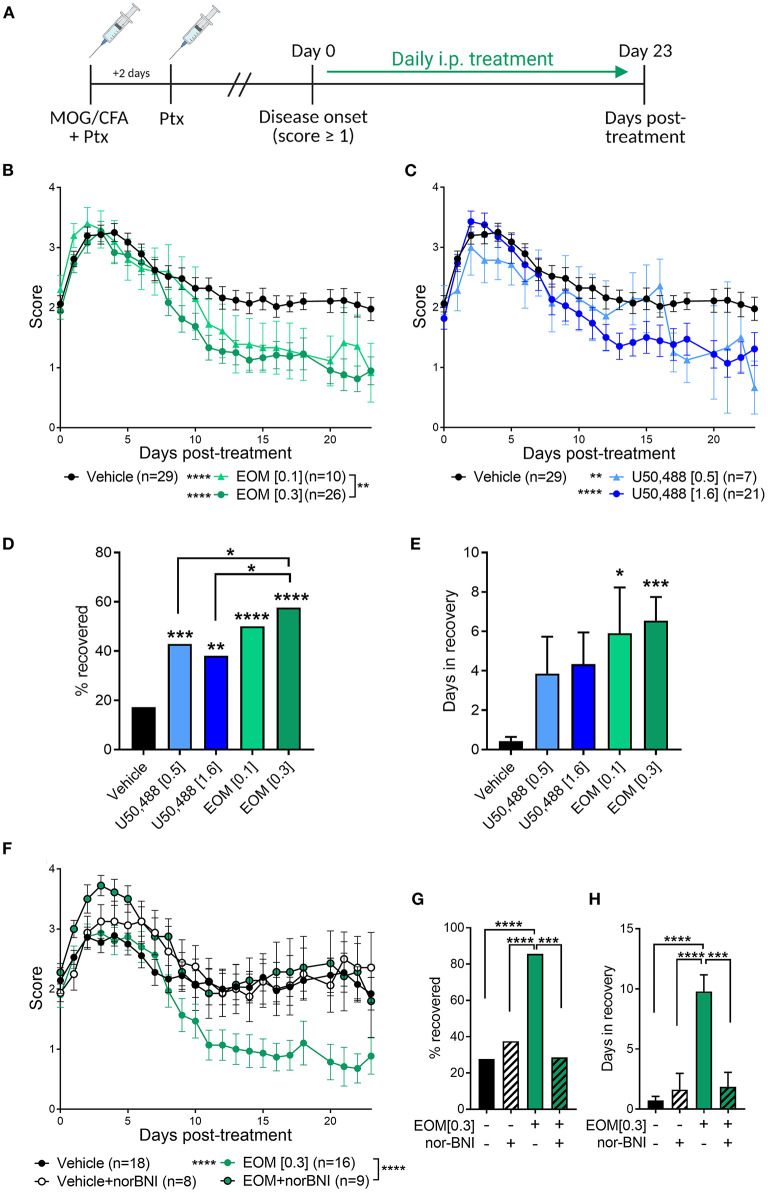
In experimental autoimmune encephalomyelitis (EAE) mice, treatment with EOM SalB was more effective than U50,488 at enabling KOR-mediated functional recovery. **(A)** Mice were immunized with myelin oligodendrocyte glycoprotein (MOG) in complete Freund's adjuvant (CFA) with pertussis toxin (Ptx) and treated daily from onset (score ≥ 1) with vehicle, EOM SalB or U50,488. **(B)** Disease score of EOM SalB or **(C)** U50,488 treated mice from disease onset to day 23. Scores aligned to the day of disease onset (day 0 post-treatment). Results are combined from 3-7 independent experiments (*n* = 7–29, as indicated). **(D)** Percentage recovery to 23 days post-treatment (recovery = score ≤ 0.5). Fisher's exact test for % recovery compared to vehicle or EOM SalB (0.3 mg/kg). **(E)** Number of days in recovery to 23 days post-treatment. **(F)** Animals treated with vehicle or KOR antagonist nor-BNI (10 mg/kg, weekly) and treated with vehicle or EOM SalB (0.3 mg/kg). Results are combined from 2 to 3 independent experiments (*n* = 8–16, as indicated). **(G)** Percentage of mice recovery by day 23. Fisher's exact test for % recovery compared to EOM SalB (0.3 mg/kg). **(H)** Number of days spent in recovery up to day 23. Two-way ANOVA test with Dunnett's multiple comparison test was used for scoring data to compare treatment doses. One-way ANOVA for days in recovery data with Tukey's multiple comparison test. Data presented as mean ± SEM. *p < 0.05, **p < 0.01, ***p < 0.001, ****p < 0.0001. Image in panel **(A)** created using Biorender.com.

EAE was used to evaluate the therapeutic effect of EOM SalB (Figure 2A). EOM SalB (0.1 and 0.3 mg/kg) showed a dose-dependent attenuation of disease score compared to vehicle (*p* < 0.0001), with the 0.3 mg/kg dose significantly more efficacious compared to the 0.1 mg/kg dose (*p* < 0.01; Figure 2B). Similarly, both doses of U50,488 (0.5 and 1.6 mg/kg) reduced disease scores compared to vehicle (*p* < 0.01; Figure 2C). However, when comparing the percentage of mice recovered (recovery defined as a score ≤ 0.5) the 0.3 mg/kg dose of EOM SalB was more beneficial in enabling recovery than both doses of U50,488 (*p* < 0.05; Figure 2D). In addition, both tested doses of EOM SalB significantly increased the number of days in recovery (days with a score ≤ 0.5) compared to vehicle (*p* < 0.05), whereas neither of the tested doses of U50,488 showed a significant increase (*p* > 0.05; Figure 2E).

The long-acting KOR antagonist, nor-BNI, was used to confirm whether the effects of EOM SalB (0.3 mg/kg) were KOR mediated. Pretreatment with nor-BNI prevented EOM SalB from enabling recovery (*p* < 0.0001), with disease scores similar to those seen in mice administered vehicle (Figure 2F). Nor-BNI also prevented EOM SalB from enabling full recovery [percentage of mice recovered (*p* < 0.001; Figure 2G)], or altering the number of days in recovery (*p* < 0.001; Figure 2H). This data suggests that EOM SalB at the tested doses was significantly better to reduce EAE severity compared to vehicle and U50,488 and that the effect of EOM SalB was KOR mediated.

### CNS-Infiltrating Immune Cells Are Reduced by EOM SalB and U50,488 Treatment in the EAE Model

**Figure 3:**
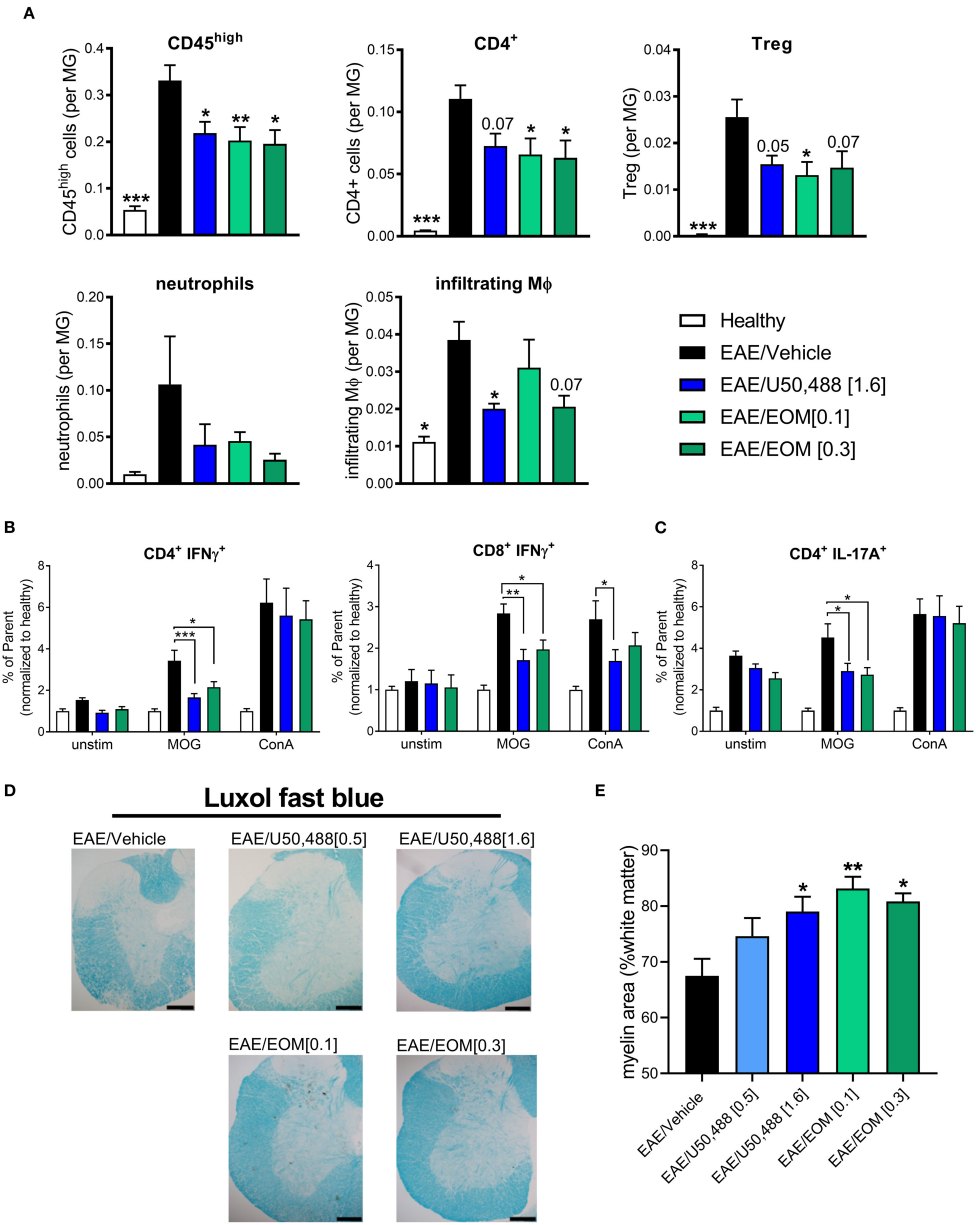
The number of CNS-infiltrating immune cells were reduced by daily KOR agonist treatment. Analysis of lymphocyte populations from healthy, vehicle, EOM SalB (0.1 or 0.3 mg/kg) and U50,488 (1.6 mg/kg) treated brain tissue. All infiltrating immune cells were identified by CD45^high^ expression and the relative number of cells is expressed as a ratio to microglia. **(A)** Infiltrating cells identified by CD45^high^ expression, CD4^+^ T cells, CD4^+^CD25^+^ regulatory T cells (Treg), neutrophils and infiltrating macrophages (MΦ). Shown are the results from 3 independent experiments with 8–12 mice per group. One-way ANOVA with Dunnett's multiple comparison test. **(B)** Splenocyte cultures were either unstimulated (unstim) or stimulated with myelin oligodendrocyte glycoprotein (MOG) or Concanavalin A (ConA). CD4^+^ and CD8^+^ T-cell intracellular interferon (IFN)γ (as frequency of parent, CD4^+^ or CD8^+^) from splenocytes of EAE animals treated daily from onset with vehicle, U50,488 (1.6 mg/kg), or EOM SalB (0.3 mg/kg). **(C)** CD4^+^ T-cell IL-17A^+^ (as frequency of parent, CD4^+^) from splenocytes of EAE animals treated daily from onset with vehicle, U50,488 (1.6 mg/kg), or EOM SalB (0.3 mg/kg). **(D)** Representative images of spinal cord sections from age-matched EAE animals treated with vehicle, U50,488 (0.5 mg/kg, 1.6 mg/kg) or EOM SalB (0.1 mg/kg, 0.3 mg/kg) until day 44. Scale bars, 100 μm. **(E)** Percentage of white matter area stained for myelin per region of interest, determined using thresholding in luxol fast blue stained spinal cord sections. Results are shown from 3 independent EAE experiments, *n* = 5–10 animals, two sections of the cervical spinal cord per animal. Kruskal-Wallis with Dunn's multiple comparison test. Data presented as mean ± SEM. *p < 0.01, **p < 0.05, ***p < 0.001.

We assessed whether EOM SalB altered the immune environment contributing to EAE disease reduction. Infiltration of immune cells (gated as CD45^high^; gating strategy in Supplementary Figure 3A) showed reduced infiltration into the brain following treatment with EOM SalB (0.1 and 0.3 mg/kg) and U50,488 (1.6 mg/kg) compared to vehicle (Figure 3A). To further assess which specific cell types were affected, we assessed CD4^+^ T cells, regulatory T cells (Tregs), neutrophils and infiltrating macrophages, as well as CD8^+^ T cells, resident macrophages, B cells, monocytes and dendritic cells (gated on the frequency of CD45^int^ microglia; gating strategy in Supplementary Figure 3B). Most significantly, the CD4^+^ T cells, Tregs, and infiltrating macrophages were reduced following treatment with both EOM SalB and U50,488 (Figure 3A) while the other cell types show only a modest reduction in the brain compared to vehicle (Supplementary Figure 3C). This suggests that one way these KOR agonists are improving EAE severity is by targeting immune cell infiltration into the CNS.

Given the previously reported immunomodulatory effects of KOR agonists, we assessed whether KOR agonist treatment altered antigen-specific T cells by re-stimulating splenocytes collected from mice that underwent the EAE model to analyze intracellular cytokines (gating strategy in Supplementary Figure 4A). A MOG-specific reduction in the percentage of IFNγ^+^ T cells (CD4^+^ or CD8^+^) following both U50,488 and EOM SalB treatment in the EAE model could be detected (Figure 3B). This is suggestive of a reduction in Th1 pro-inflammatory response. Similarly, there was a reduction in the percentage of IL-17A^+^CD4^+^ T cells (Figure 3C), which is suggestive of a reduced Th17 pro-inflammatory response following both U50,488 and EOM SalB treatment in the EAE model. There were no differences in the percentage of CD4^+^ and CD8^+^ T cells, IL-17A^+^CD8^+^ T-cells, or the MFI of IL-10^+^CD4^+^ or CD8^+^ T cells from splenocytes of EAE animals treated daily from the onset with vehicle, U50,488 (1.6 mg/kg), or EOM (0.3 mg/kg) (Supplementary Figures 4B,D). Interestingly, the significant effects observed were in MOG re-stimulated cells, highlighting the specific sensitization of these cells toward the MOG peptide. This data suggests that the effect of EOM SalB on immune cells is shifting the T cell response away from a Th1 and Th17 pro-inflammatory environment.

We further sought to understand the effects of the treatments on myelination in spinal cords collected from mice with EAE. Assessment of the myelin levels with luxol fast blue staining found that there was increased myelin following EOM SalB (0.1 and 0.3 mg/kg) and U50,488 (1.6 mg/kg) treatment compared to vehicle (*p* < 0.05; Figures 3D,E). However, H&E staining in the spinal cord was used to assess immune cell infiltration, with no significant differences observed between treatment groups (Supplementary Figure 5). Overall, this data shows that reduced EAE severity after treatment with EOM SalB is mediated by reduced infiltration of immune cells into the CNS, shifting of the immune environment and increased myelination.

### EOM SalB Attenuated Weight Loss in Cuprizone-Treated Mice

**Figure 4:**
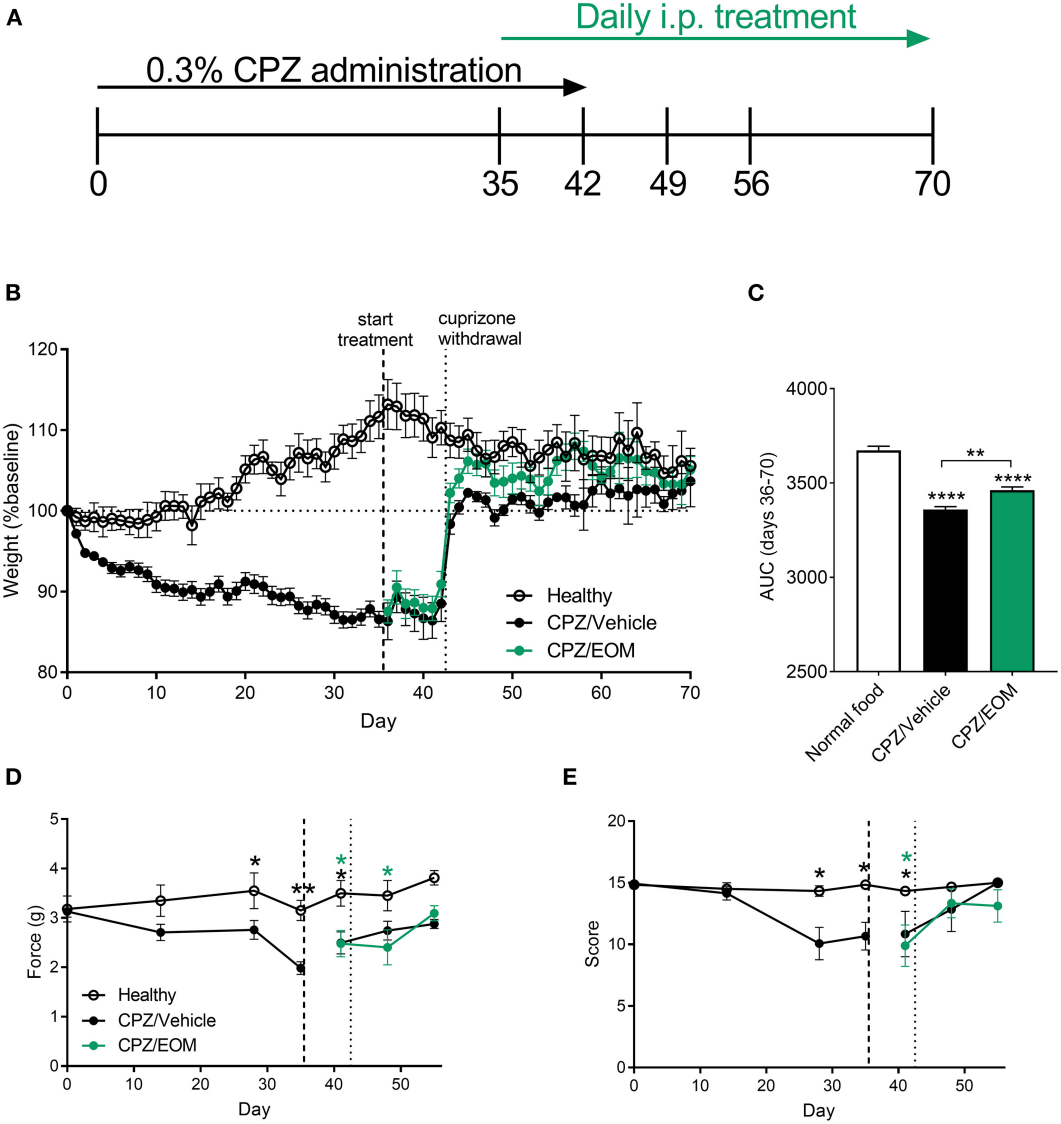
**(A)** Mice were administered 0.3% cuprizone (CPZ) on days 0–42 and daily EOM SalB treatment on days 35-70. **(B,C)** EOM SalB (0.3 mg/kg/i.p./day)-treated mice gained significantly more weight than vehicle-treated mice [area under the curve (AUC) analysis of the weights on days 36–70)]. **(D)** Cuprizone administration significantly decreased the force paw-withdrawal threshold for mechanical stimulation, however, EOM SalB (0.3 mg/kg) treatment showed no significant change compared to vehicle-treated mice. **(E)** Cuprizone administration significantly decreased the score on the horizontal bar test, however, EOM (0.3 mg/kg) treatment had no significant effect compared to vehicle-treated mice. **(D,E)** Two-way repeated-measures ANOVA with Bonferroni post-tests (separate analysis for days 0–35 and days 36–56). **(C)** One-way ANOVA with Bonferroni post-tests. Data presented as mean ± SEM. ^*^p < 0.05, ^**^p < 0.01, ^***^p < 0.001, ^****^p < 0.0001. *n* = 6–9 per treatment group. Image in panel **(A)** created using Biorender.com.

To understand the effect of EOM SalB on demyelination and remyelination without the influence of immune cells, demyelination was induced by cuprizone leading to reduced myelin in the brain. During cuprizone-induced demyelination (Figure 4A), mice were weighed daily as a measure of general health. Cuprizone administration lead to a significant weight loss compared to baseline (day 0) (Figure 4B), and mice administered cuprizone lost more weight than the healthy mice. The area under the curve analysis from EOM SalB (0.3 mg/kg) treated mice (days 36–70) showed a significant increase in weight compared to the vehicle-treated group (Figure 4C).

In addition, two behavioral assays to measure mechanical sensitivity and motor coordination were performed to assess whether EOM SalB was able to enhance functional recovery. It was found there was a significant decrease in the mechanical withdrawal threshold (Figure 4D) and motor coordination score (Figure 4E) with cuprizone administration; however, EOM SalB treatment was not able to reverse these effects.

### EOM SalB Significantly Increased the Number of Oligodendrocytes in the Corpus Callosum

**Figure 5:**
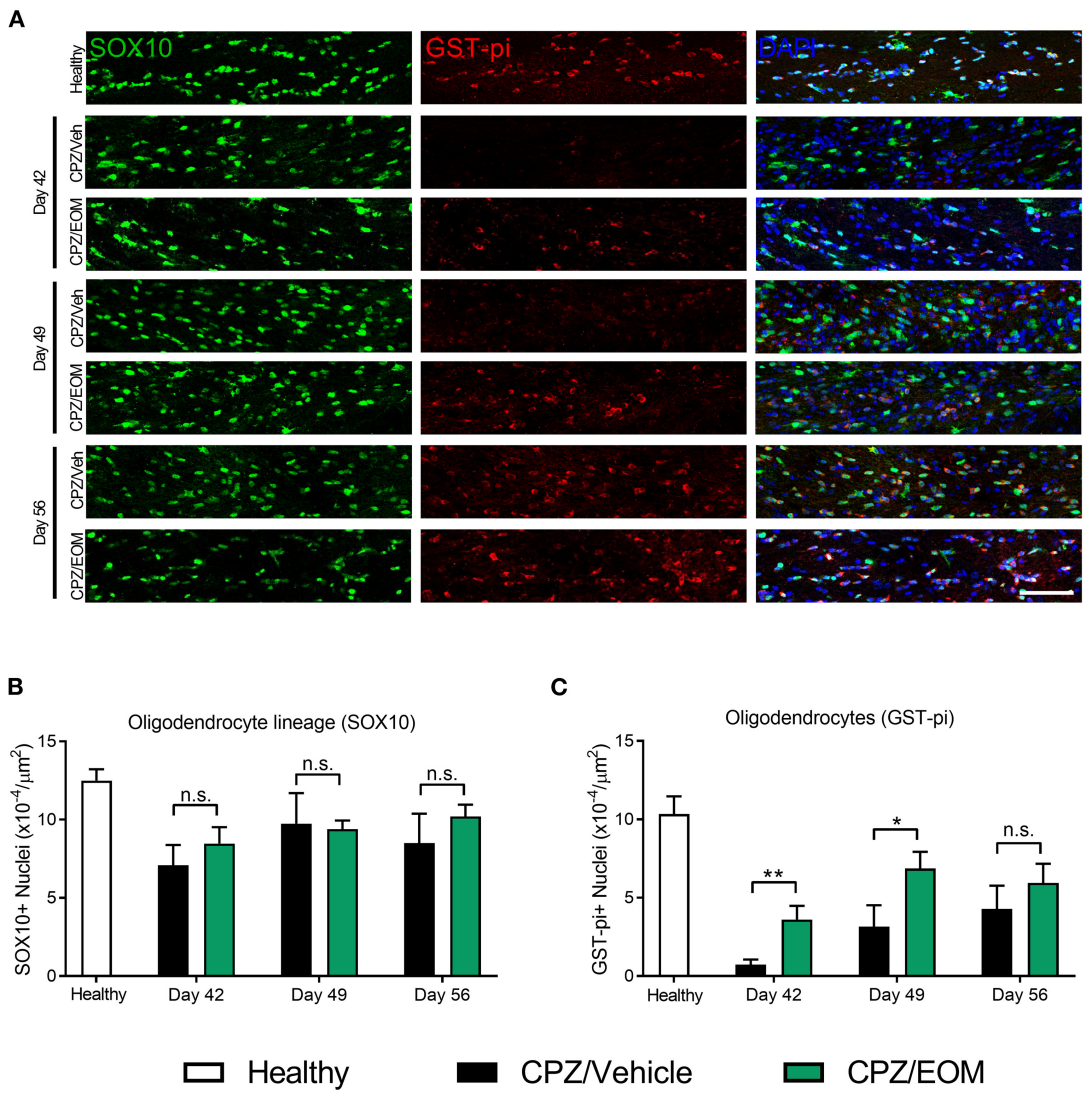
EOM SalB administration changes the number of oligodendrocytes in the corpus callosum of mice administered 0.3% cuprizone (CPZ). **(A)** Representative images showing DAPI-positive nuclei (blue), SOX10-positive cells identifying all cells within the oligodendrocyte lineage (green) and mature GST-pi-positive mature oligodendrocytes (red) in the region of interest. Scale bar is 100 μm. **(B)** The number of SOX10-positive cells was not significantly altered following EOM SalB treatment (0.3 mg/kg). **(C)** EOM SalB administration increased the number of GST-pi-positive oligodendrocyte cells at days 42 and 49 compared to the vehicle-treated control mice. Images were analyzed using Cell Profiler in 3 sections per animal (averaged), *n* = 5–6 mice per treatment. Unpaired t-test. Data presented as mean ± SEM. *p < 0.05, **p < 0.01, n.s. not significant.

The brains of mice from the cuprizone-induced demyelination model were collected at days 42, 49, and 56 to assess the number of SOX10-positive (oligodendrocyte lineage cells) and GST-pi-positive cells (oligodendrocytes) in the corpus callosum (Figure 5A). At each of the time points, it was found that there was no significant difference in the number of SOX10-positive cells when EOM SalB (0.3 mg/kg) treatment was compared to vehicle (*p* > 0.05, Figure 5B). However, when comparing the number of GST-pi-positive cells, there was a significant increase in the number found in the mice treated with EOM SalB compared to vehicle at day 42 and 49 (*p* < 0.05; Figure 5C), suggesting a significant increase of mature oligodendrocytes in these animals.

### EOM SalB Enhanced Remyelination in the Corpus Callosum

**Figure 6:**
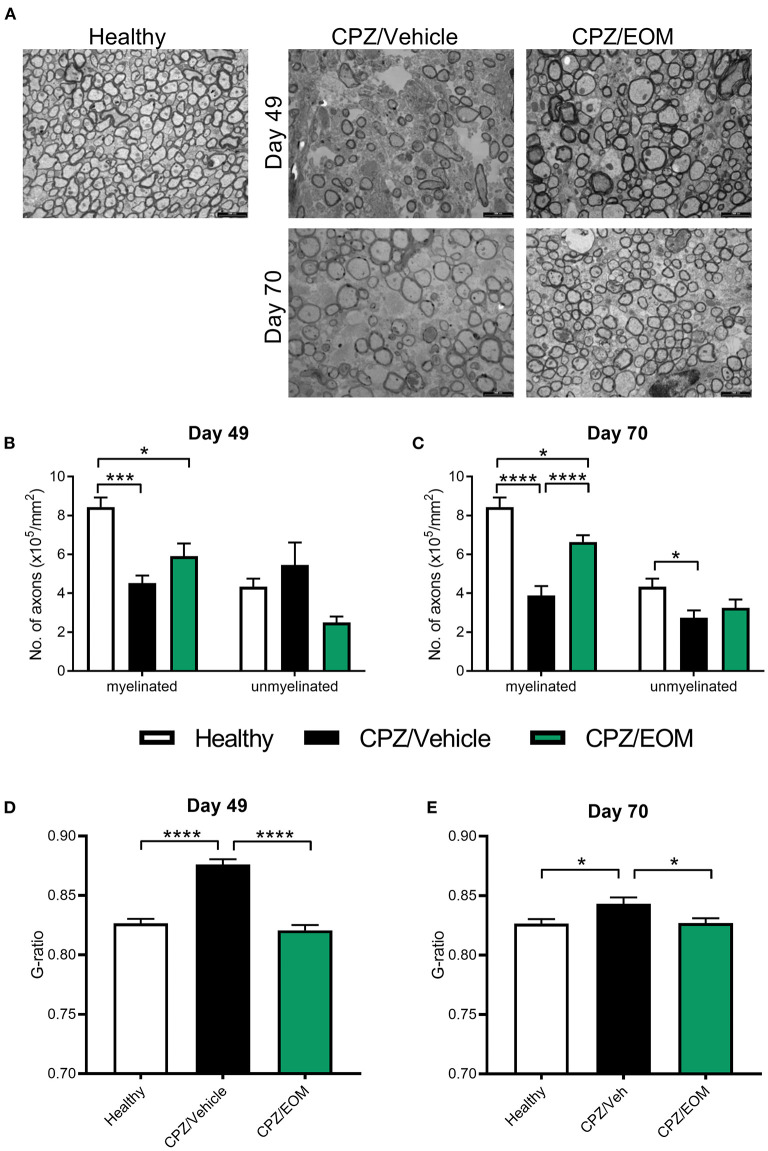
EOM SalB treatment increased remyelination within the corpus callosum of mice administered 0.3% cuprizone. **(A)** Representative transmission electron microscopy images showing myelinated and unmyelinated axons in the corpus callosum of healthy and mice administered cuprizone (CPZ) and treated with either vehicle (Veh) or EOM SalB (0.3 mg/kg). The number of myelinated and unmyelinated axons were counted at **(B)** day 49 and **(C)** day 70. EOM SalB (0.3 mg/kg) treatment led to an increase in the number of myelinated axons at day 70. Two-way ANOVA with Tukey's multiple comparisons test. The g-ratio of each axon was calculated by dividing the inner axonal diameter by the outer diameter at **(D)** day 49 and **(E)** day 70. EOM SalB treatment decreased the g-ratio to healthy levels at days 49 and 70. One-way ANOVA with Tukey's multiple comparisons test. Five images per animal, three animals per treatment group. Scale bar is 2,000 nm. Data presented as mean ± SEM. *p < 0.05, **p < 0.01, ***p < 0.001, ****p < 0.0001.

To test whether the increase in oligodendrocyte numbers reflects increased remyelination, the corpus callosum from cuprizone-treated mice were assessed for remyelination using TEM at days 49 and 70 (Figure 6A). On day 49, there was a decrease in the number of myelinated axons in the mice that were administered cuprizone compared to the healthy mice (*p* < 0.05, Figure 6B). There was no change in the number of unmyelinated axons at day 49 (Figure 6B). On day 70, EOM SalB treatment significantly increased the number of myelinated axons compared to vehicle, however, did not affect the number of unmyelinated axons (Figure 6C). We used g-ratios to assess the myelin thickness, at both time points. Cuprizone-intoxicated mice treated with vehicle had significantly increased g-ratios compared to healthy controls (*p* < 0.05), and treatment of EOM SalB (0.3 mg/kg) lead to a rapid reduction in the g-ratio to the same level as healthy mice at both day 49 and 70 (Figures 6D,E).

## Discussion

There is an urgent need to develop remyelination treatments to reduce the burden of disease in demyelinating diseases such as MS (3, 31), optic neuritis (32, 33) and other diseases where myelin is damaged including Alzheimer's (34) and Parkinson's disease (35). The key to developing these therapeutics lies in identifying safe therapeutic targets that enable myelin repair and restoration of lost functions (36). The KOR has been identified as a potential target for remyelination (4, 5, 37, 38). Unfortunately, the KOR agonist evaluated in these studies, U50,488 has side effects including sedation and aversion that limit its clinical use (7–11). In contrast, the KOR agonist nalfurafine has proven that some KOR agonists can be clinically safe (39). We aimed to explore the Salvinorin A structural class of KOR agonists for its ability to reduce disease and promote remyelination and repair in preclinical models of MS. The KOR agonist EOM SalB was selected for its potency, selectivity and reduced side-effect profile (18, 19). In the current study, we have shown that treatment with EOM SalB in the two different mouse models of MS showed a significant recovery in myelination as well as reduced cellular infiltration.

There is substantial evidence suggesting that G-protein biased KOR agonists are associated with fewer β-arrestin associated side effects (40–43). Calculated G-protein bias values vary due to a range of different factors, including the use of different reference ligands, cell types, signaling assays and species of the receptor (44). In the present study, we have shown that EOM SalB has a bias factor of 2.53 compared to the unbiased reference ligand U50,488. This is consistent with a previous study, showing that EOM SalB was G-protein biased using the GloSensor luciferase-based assay in HEK293T cells to measure cAMP and the Tango assay to measure β-arrestin recruitment in HTLA cells (45). The bias factor in this study was 15.26 with U50,488 as the reference ligand (45). This provides confidence in our data showing that EOM Sal B is G-protein biased and that these G-protein bias results are reproducible across different cell types and signaling assays.

The confirmation that EOM SalB is G-protein biased is also consistent with the reduced side effect profile. In Sprague-Dawley rats, we have previously shown that EOM SalB (0.1–0.3 mg/kg) was not sedative in the spontaneous locomotor activity test, did not show anxiogenic behavior in the elevated plus-maze, did not have depressive-like effects in the forced swim test, and did not show aversion in the conditioned place aversion test (0.1 mg/kg) (19). However, in C57Bl/6J mice, Kaski et al. (45) found that a 1 mg/kg dose of EOM SalB produced aversion in the conditioned placed aversion test, impaired motor coordination in the rotarod performance test and reduced novelty-induced locomotion. These differences in side effects are likely to be due to the increased dose used by Kaski et al. (45) as well as the different species used. Nonetheless, it does appear from these results that the therapeutic window for EOM SalB would be at a dose >1 mg/kg and that the dose of 0.1–0.3 mg/kg is well tolerated.

Using the EAE model, we demonstrated that EOM SalB (0.1–0.3 mg/kg) reduced disease score and increased recovery at greater rates than U50,488 (0.5–1.6 mg/kg) when administered therapeutically. Additionally, this effect was KOR-mediated as the effect was reversed with the KOR antagonist nor-BNI. Our evaluation of EOM SalB in EAE is the first study to assess the Salvinorin structural class of KOR agonists for their ability to promote disease recovery *in vivo*. Furthermore, we found that EOM SalB and U50,488 treatment increased the levels of myelin staining using luxol fast blue indicating enhanced remyelination.

Our data are consistent with previous findings that KOR agonists reduce EAE disease. Du et al. (4) originally showed that KOR knockout mice were more susceptible to EAE, which was not apparent when either the mu or delta opioid receptor were knocked out. Furthermore, the study demonstrated that prophylactic treatment with U50,488 (0.5–5 mg/kg) reduced EAE severity. Similarly, Tangherlini et al. (6) showed that prophylactic administration of quinoxaline-based KOR agonists reduced EAE severity in a KOR-dependent fashion. Denny et al. (22) showed that therapeutic KOR agonist administration, where treatment starts after the initial onset of symptoms, led to a decrease in EAE disease scores, using both U50,488 (1.6 mg/kg) and nalfurafine (0.0003–0.1 mg/kg). This therapeutic administration tests the ability of KOR agonists to induce recovery rather the prophylactic treatment regimen that tests a combination of disease induction, progression and recovery. Therapeutic administration models a clinically relevant treatment strategy.

We assessed whether EOM SalB altered the immune environment contributing to EAE disease reduction. CNS inflammation associated with MS and the EAE model is regulated by many cell types including T cells, B cells, neutrophils, monocytes, macrophages, and resident microglia (46). We found a reduced infiltration of CD45^high^ immune cells including CD4^+^ T cells, Tregs, and infiltrating macrophages following treatment with both EOM SalB and U50,488. We further assessed the effect of KOR agonist treatment on antigen-specific T cell responses by re-stimulating splenocytes to analyze intracellular cytokines. We identified a MOG-specific reduction in IFNγ^+^CD4^+^ and CD8^+^ T cells as well as a reduction in IL-17A^+^CD4^+^ T cells with KOR agonist treatment, suggesting that EOM SalB treatment not only targets the infiltration of immune cells into the CNS but also shifts the balance from a Th1 and Th17 inflammatory cytokine response toward a favorable regulatory environment. The immunomodulatory properties of KOR have been previously explored, suggesting that the kappa opioid system can interact with the immune system and is consistent with Tangherlini et al. (6) and Denny et al. (22), with both studies showing a reduction in CD45^+^ cells following KOR agonist treatment, with a corresponding reduction in IFNγ and IL-17A. Given that both IFNγ-producing Th1 and IL-17A-producing Th17 are critical drivers of the immune-mediated demyelination in MS, this finding suggests that KOR agonist treatment not only enhances remyelination but also reduces the myelin-damaging immune response.

Cuprizone is a copper chelating neurotoxin with region-dependent toxicity, selectively causing oligodendrocyte cell death, activation of astrocytes and microglia, leading to demyelination (23, 24). As opposed to the EAE model, T cells are not believed to play a major role in the disease progression in the cuprizone-induced demyelination model (47). Therefore, it is believed the model better represents the progressive forms of MS and enables study into the effect of treatment in a different demyelination setting. The cuprizone-induced demyelination model is widely used, however, less is known about the associated behavioral deficits. A recent review found that approximately 29–86% of MS patients experience pain, while only 4% of papers using the cuprizone model tested this behavior (48). Therefore, we evaluated neuropathic pain using mechanical sensitivity and function using motor coordination, which is a more commonly assessed measure in the cuprizone model.

Our results show the behaviors of the cuprizone–treated mice are significantly different from healthy controls but once cuprizone is removed from the diet, the cuprizone-induced behaviors rapidly return to healthy levels resulting in a very limited window to observe potential therapeutic effects. Treatment with EOM SalB showed no difference to vehicle-treated mice. Motor behavior assays have been used in previous cuprizone studies (48, 49); however, evoked tests may not have the sensitivity to detect subtle alterations in behavior. For a more robust behavioral deficit, a more robust model of demyelination may be required such as the augmented cuprizone model whereby rapamycin, an mTOR inhibitor, is administered daily during cuprizone treatment to prevent spontaneous remyelination and allowing evaluation of therapeutic treatments over an extended period of time (50, 51). Additionally, a more complex behavioral model may be able to detect these deficits, such as the mouse motor skill sequence (MOSS) activity wheel. This non-evoked model requires communication between both hemispheres in the brain for complex bilateral motor coordination, which is an essential role of the corpus callosum (52, 53). However, complex MOSS behaviors are yet to be utilized to assess therapeutics in this model.

The cuprizone-induced demyelination model was used to understand the effects of EOM SalB on remyelination. The administration of cuprizone is known to specifically cause oligodendrocyte apoptosis which leads to demyelination (23, 24). For remyelination to occur, OPCs must migrate to the site of injury and differentiate into mature oligodendrocytes capable of remyelinating damaged axons. We used a marker for oligodendrocyte lineage cells (SOX10), which encompasses OPCs and oligodendrocytes, showing that the lineage cell numbers were not altered with the administration of EOM SalB. However, we found that there was an increase in the number of GST-pi-positive oligodendrocyte cells, indicating that EOM SalB is not altering the number of OPCs migrating to the corpus callosum but is specifically altering the number of mature oligodendrocytes. It has previously been shown that KOR agonists are capable of differentiating OPCs to mature oligodendrocytes *in vitro* (4, 5). Mei et al. (5) showed U50,488 treatment caused differentiation of purified rat OPC cultures into myelin basic protein (MBP)-positive oligodendrocytes, the effect of which was abolished in KOR null cultures. Furthermore, Mei et al. used human induced pluripotent stem cell-derived OPC cultures and showed U50,488 induced OPC differentiation into mature oligodendrocytes. While this supports OPC maturation, these effects may be due to many factors as KOR is located on all key cell types that enable effective remyelination (54–57). We know that cuprizone administration also significantly activates astrocytes and microglia (58), however, the role of KOR on these cell types is unknown.

To accurately quantify myelin, we used TEM to examine myelin thickness in cuprizone-administered mice. Our data showed that EOM SalB (0.3 mg/kg) treatment, lead to an increase in the number of myelinated axons at day 70, and a decrease in the g-ratio to healthy levels by day 49, which was also seen at day 70. We have previously shown that nalfurafine treatment until day 70 in the cuprizone model led to an increase in the percentage of myelinated axons and a decrease in the g-ratio compared to vehicle in the corpus callosum (22). This data shows that KOR agonists are capable of inducing remyelination in the cuprizone-induced demyelination model.

Overall, our results show that EOM SalB is a G-protein biased KOR agonist that effectively reduces EAE disease severity in a KOR-dependent manner, with a greater percentage of mice recovered than U50,488. EOM SalB reduced the number of infiltrating immune cells in the CNS and increased the myelin in EAE mice. In the cuprizone-induced demyelination model, EOM SalB treatment rapidly increased the number of oligodendrocytes in the corpus callosum, which correlated to an increase in the myelin thickness and an increase in the number of myelinated axons. This study further provides evidence that KOR agonists are effective in supporting remyelination and could be a promising target for future therapeutic development for demyelinating diseases.

## Data Availability Statement

The raw data supporting the conclusions of this article will be made available by the authors, without undue reservation.

## Ethics Statement

The animal study was reviewed and approved by the Victoria University of Wellington Animal Ethics Committee.

## Author Contributions

BK, AL, TP, KP, and NT contributed to the design of the study. TP and DL provided the kappa opioid receptor agonists. KP, KR, NT, LD, AA, AL, and DL conducted the experiments and performed the data analysis. KP wrote the first draft of the manuscript. BK, AL, TP, and KR critically evaluated the manuscript. All authors contributed to the manuscript revision, read, and approved the submitted version.

## Funding

This study was supported by funding from the Neurological Foundation of New Zealand (#1639PG to BK, AL, and TP), the Health Research Council of New Zealand (#18/063 to BK, AL, and TP), the National Institutes of Health (RO1DA018151 to TP), the Ministry of Business, Innovation and Employment (RTVU1802 to AL and BK), and the Great New Zealand Trek Charitable Trust (to AL).

## Conflict of Interest

The authors declare the following potential conflicts of interest with respect to the research, authorship and/or publication of this article: AL, BK, and TP are inventors on patent applications that relate to this work and have been licensed to Rekover Therapeutics Ltd. AL, BK, and TP are founding scientists of Rekover Therapeutics Ltd. The remaining authors declare that the research was conducted in the absence of any commercial or financial relationships that could be construed as a potential conflict of interest.

## Publisher's Note

All claims expressed in this article are solely those of the authors and do not necessarily represent those of their affiliated organizations, or those of the publisher, the editors and the reviewers. Any product that may be evaluated in this article, or claim that may be made by its manufacturer, is not guaranteed or endorsed by the publisher.

## References

[1] Walton C King R Rechtman L Kaye W Leray E Marrie RA. Rising prevalence of multiple sclerosis worldwide: Insights from the Atlas of MS, third edition. Mult Scler. (2020) 26:1816–21. 10.1177/1352458520970841 33174475 PMC7720355

[2] McGinley MP Goldschmidt CH Rae-Grant AD. Diagnosis and treatment of multiple sclerosis: a review. JAMA. (2021) 325:765–79. 10.1001/jama.2020.26858 33620411

[3] Lubetzki C Zalc B Williams A Stadelmann C Stankoff B. Remyelination in multiple sclerosis: from basic science to clinical translation. Lancet Neurol. (2020) 19:678–88. 10.1016/S1474-4422(20)30140-X 32702337

[4] Du C Duan Y Wei W Cai Y Chai H Lv J. Kappa opioid receptor activation alleviates experimental autoimmune encephalomyelitis and promotes oligodendrocyte-mediated remyelination. Nat Commun. (2016) 7:11120. 10.1038/ncomms11120 27040771 PMC4822006

[5] Mei F Mayoral SR Nobuta H Wang F Desponts C Lorrain DS. Identification of the kappa-opioid receptor as a therapeutic target for oligodendrocyte remyelination. J Neurosci 36(30). (2016) 7925–35. 10.1523/JNEUROSCI.1493-16.2016 27466337 PMC4961778

[6] Tangherlini G Kalinin DV Schepmann D Che T Mykicki N Stander S. Development of novel quinoxaline-based kappa-opioid receptor agonists for the treatment of neuroinflammation. J Med Chem. (2019) 62:893–907. 10.1021/acs.jmedchem.8b01609 30543421

[7] Mucha RF Herz A. Motivational properties of kappa and mu opioid receptor agonists studied with place and taste preference conditioning. Psychopharmacology (Berl). (1985) 86:274–80. 10.1007/BF00432213 2994144

[8] Ehrich JM Messinger DI Knakal CR Kuhar JR Schattauer SS Bruchas MR. Kappa opioid receptor-induced aversion requires p38 MAPK activation in VTA dopamine neurons. J Neurosci. (2015) 35:12917–31. 10.1523/JNEUROSCI.2444-15.2015 26377476 PMC4571610

[9] Zhang LS Wang J Chen JC Tao YM Wang YH Xu XJ. Novel kappa-opioid receptor agonist MB-1C-OH produces potent analgesia with less depression and sedation. Acta Pharmacol Sin. (2015) 36:565–71. 10.1038/aps.2014.145 25816912 PMC4422940

[10] Wang YJ Hang A Lu YC Long Y Zan GY Li XP. kappa Opioid receptor activation in different brain regions differentially modulates anxiety-related behaviors in mice. Neuropharmacology. (2016) 110:92–101. 10.1016/j.neuropharm.2016.04.022 27106167

[11] Paton KF Atigari DV Kaska S Prisinzano T Kivell BM. Strategies for developing kappa opioid receptor agonists for the treatment of pain with fewer side effects. J Pharmacol Exp Ther. (2020) 375:332–48. 10.1124/jpet.120.000134 32913006 PMC7589957

[12] Roth BL Baner K Westkaemper R Siebert D Rice KC Steinberg S. Salvinorin A: a potent naturally occurring nonnitrogenous kappa opioid selective agonist. Proc Natl Acad Sci U S A. (2002) 99:11934–9. 10.1073/pnas.182234399 12192085 PMC129372

[13] Kivell BM Ewald AW Prisinzano TE. Salvinorin A analogs and other kappa-opioid receptor compounds as treatments for cocaine abuse. Adv Pharmacol. (San Diego, Calif). (2013) 69:481–511. 10.1016/B978-0-12-420118-7.00012-3 24484985 PMC4128345

[14] Morani AS Ewald A Prevatt-Smith KM Prisinzano TE Kivell BM. The 2-methoxy methyl analogue of salvinorin A attenuates cocaine-induced drug seeking and sucrose reinforcements in rats. Eur J Pharmacol. (2013) 720:69–76. 10.1016/j.ejphar.2013.10.050 24201308 PMC3899895

[15] Zjawiony JK Machado AS Menegatti R Ghedini PC Costa EA Pedrino GR. Cutting-edge search for safer opioid pain relief: retrospective review of salvinorin A and its analogs. Front Psychiatry. (2019) 10:157. 10.3389/fpsyt.2019.00157 30971961 PMC6445891

[16] Paton KF Biggerstaff A Kaska S Crowley RS La Flamme AC Prisinzano TE. Evaluation of biased and balanced salvinorin a analogs in preclinical models of pain. Front Neurosci. (2020) 14:765. 10.3389/fnins.2020.00765 32792903 PMC7385413

[17] Munro T.A. Duncan K.K. Xu W. Wang Y. Liu-Chen L.Y. Carlezon W.A. Jr. (2008). Standard protecting groups create potent and selective kappa opioids: salvinorin B alkoxymethyl ethers. Bioorg Med Chem. 16: 1279–86. 10.1016/j.bmc.2007.10.067 17981041 PMC2568987

[18] Prevatt-Smith KM Lovell KM Simpson DS Day VW Douglas JT Bosch P. Potential drug abuse therapeutics derived from the hallucinogenic natural product salvinorin A. Medchemcomm. (2011) 2:1217–22. 10.1039/c1md00192b 22442751 PMC3307802

[19] Ewald AWM Bosch PJ Culverhouse A Crowley RS Neuenswander B Prisinzano TE. The C-2 derivatives of salvinorin A, ethoxymethyl ether Sal B and beta-tetrahydropyran Sal B, have anti-cocaine properties with minimal side effects. Psychopharmacology (Berl). (2017) 234:2499–514. 10.1007/s00213-017-4637-2 28536865 PMC5542847

[20] Bruchas MR Chavkin C. Kinase cascades and ligand-directed signaling at the kappa opioid receptor. Psychopharmacology (Berl). (2010) 210:137–47. 10.1007/s00213-010-1806-y 20401607 PMC3671863

[21] Schattauer SS Kuhar JR Song A Chavkin C. Nalfurafine is a G-protein biased agonist having significantly greater bias at the human than rodent form of the kappa opioid receptor. Cell Signal. (2017) 32:59–65. 10.1016/j.cellsig.2017.01.016 28088389 PMC5779083

[22] Denny L Al Abadey A Robichon K Templeton N Prisinzano TE Kivell BM. Nalfurafine reduces neuroinflammation and drives remyelination in models of CNS demyelinating disease. Clini Translat Immunol. (2021) 10:e1234. 10.1002/cti2.1234 33489124 PMC7811802

[23] Matsushima GK Morell P. The neurotoxicant, cuprizone, as a model to study demyelination and remyelination in the central nervous system. Brain Pathol. (2001) 11:107–16. 10.1111/j.1750-3639.2001.tb00385.x 11145196 PMC8098267

[24] Praet J Guglielmetti C Berneman Z Van der Linden A Ponsaerts P. Cellular and molecular neuropathology of the cuprizone mouse model: clinical relevance for multiple sclerosis. Neurosci Biobehav Rev. (2014) 47:485–505. 10.1016/j.neubiorev.2014.10.004 25445182

[25] Riley AP Groer CE Young D Ewald AW Kivell BM Prisinzano TE. Synthesis and kappa-opioid receptor activity of furan-substituted salvinorin A analogues. J Med Chem. (2014) 57:10464–75. 10.1021/jm501521d 25426797 PMC4281103

[26] Crowley RS Riley AP Sherwood AM Groer CE Shivaperumal N Biscaia M. Synthetic studies of neoclerodane diterpenes from salvia divinorum: identification of a potent and centrally acting mu opioid analgesic with reduced abuse liability. J Med Chem. (2016) 59:11027–38. 10.1021/acs.jmedchem.6b01235 27958743 PMC5189922

[27] Kivell BM Paton KF Kumar N Morani AS Culverhouse A Shepherd A. Kappa opioid receptor agonist mesyl Sal B attenuates behavioral sensitization to cocaine with fewer aversive side-effects than salvinorin A in rodents. Molecules. (2018) 23:2602. 10.3390/molecules23102602 30314288 PMC6222496

[28] Templeton N Kivell B McCaughey-Chapman A Connor B La Flamme AC. Clozapine administration enhanced functional recovery after cuprizone demyelination. PLoS ONE. (2019) 14:e0216113. 10.1371/journal.pone.0216113 31071102 PMC6508663

[29] Deacon RM Thomas CL Rawlins JN Morley BJ. A comparison of the behavior of C57BL/6 and C57BL/10 mice. Behav Brain Res. (2007) 179:239–47. 10.1016/j.bbr.2007.02.009 17339058

[30] Deacon RM. Measuring motor coordination in mice. J Vis Exp. (2013) (75):e2609. 10.3791/2609 23748408 PMC3724562

[31] Villoslada P Steinman L. New targets and therapeutics for neuroprotection, remyelination and repair in multiple sclerosis. Expert Opin Investig Drugs. (2020) 29:443–59. 10.1080/13543784.2020.1757647 32299268

[32] Kawachi I. Clinical characteristics of autoimmune optic neuritis. Clinical and Experimental Neuroimmunology. (2017) 8:8–16. 10.1111/cen3.12354

[33] Andorra M Alba-Arbalat S Camos-Carreras A Gabilondo I Fraga-Pumar E Torres-Torres R. Using acute optic neuritis trials to assess neuroprotective and remyelinating therapies in multiple sclerosis. JAMA Neurol. (2020) 77:234–44. 10.1001/jamaneurol.2019.3283 31566686 PMC6777247

[34] Sun J Zhou H Bai F Zhang Z Ren Q. Remyelination: a potential therapeutic strategy for alzheimer's disease? J Alzheimers Dis. (2017) 58:597–612. 10.3233/JAD-170036 28453483

[35] Dean D .C. 3rd Sojkova J Hurley S. Kecskemeti S Okonkwo O Bendlin B.B.. (2016). Alterations of myelin content in parkinson's disease: a cross-sectional neuroimaging study. PLoS ONE. 11:e0163774. 10.1371/journal.pone.0163774 27706215 PMC5051727

[36] Ogata T. (2019). Therapeutic Strategies for Oligodendrocyte-Mediated Remyelination. In: Sango K Yamauchi J Ogata T Susuki K editors. Myelin: Basic and Clinical Advances, eds. Singapore: Springer Singapore. p. 265–279. 10.1007/978-981-32-9636-7_17 31760650

[37] Wang F Mei F. Kappa opioid receptor and oligodendrocyte remyelination. Vitam Horm. (2019) 111:281–97. 10.1016/bs.vh.2019.05.004 31421704

[38] Dworsky-Fried Z Chadwick CI Kerr BJ Taylor AMW. Multiple sclerosis and the endogenous opioid system. Front Neurosci. (2021) 15:1213. 10.3389/fnins.2021.741503 34602975 PMC8484329

[39] Kumagai H Ebata T Takamori K Muramatsu T Nakamoto H Suzuki H. Effect of a novel kappa-receptor agonist, nalfurafine hydrochloride, on severe itch in 337 haemodialysis patients: a Phase III, randomized, double-blind, placebo-controlled study. Nephrol Dial Transplant. (2010) 25:1251–7. 10.1093/ndt/gfp588 19926718

[40] White KL Robinson JE Zhu H DiBerto JF Polepally PR Zjawiony JK. The G protein-biased kappa-opioid receptor agonist RB-64 is analgesic with a unique spectrum of activities in vivo. J Pharmacol Exp Ther. (2015) 352:98–109. 10.1124/jpet.114.216820 25320048 PMC4279099

[41] Dunn AD Reed B Guariglia C Dunn AM Hillman JM Kreek MJ. Structurally related kappa opioid receptor agonists with substantial differential signaling bias: neuroendocrine and behavioral effects in C57BL6 mice. Int J Neuropsychopharmacol. (2018) 21:847–57. 10.1093/ijnp/pyy034 29635340 PMC6119295

[42] Mores KL Cummins BR Cassell RJ van Rijn RM. A review of the therapeutic potential of recently developed g protein-biased kappa agonists. Front Pharmacol. (2019) 10:407. 10.3389/fphar.2019.00407 31057409 PMC6478756

[43] Bedini A Di Cesare Mannelli L Micheli L Baiula M Vaca G De Marco R. Functional selectivity and antinociceptive effects of a novel KOPr agonist. Front Pharmacol. (2020) 11:188. 10.3389/fphar.2020.00188 32210803 PMC7066533

[44] Michel MC Charlton SJ. Biased agonism in drug discovery-is it too soon to choose a path? Mol Pharmacol. (2018) 93:259–65. 10.1124/mol.117.110890 29326242

[45] Kaski S.W. White A.N. Gross J.D. Trexler K.R. Wix K. Harland A.A.. (2019). Preclinical testing of nalfurafine as an opioid-sparing adjuvant that potentiates analgesia by the mu opioid receptor-targeting agonist morphine. J Pharmacol Exp Ther.118:255661. 10.1124/jpet.118.255661 31492823 PMC6863463

[46] Constantinescu CS Farooqi N O'Brien K Gran B. Experimental autoimmune encephalomyelitis (EAE) as a model for multiple sclerosis (MS). Br J Pharmacol. (2011) 164:1079–106. 10.1111/j.1476-5381.2011.01302.x 21371012 PMC3229753

[47] Hiremath MM Chen VS Suzuki K Ting JP Matsushima GK. MHC class II exacerbates demyelination in vivo independently of T cells. J Neuroimmunol. (2008) 203:23–32. 10.1016/j.jneuroim.2008.06.034 18805594 PMC2913406

[48] Sen MK Mahns DA Coorssen JR Shortland PJ. Behavioural phenotypes in the cuprizone model of central nervous system demyelination. Neurosci Biobehav Rev. (2019) 107:23–46. 10.1016/j.neubiorev.2019.08.008 31442519

[49] Franco-Pons N Torrente M Colomina MT Vilella E. Behavioral deficits in the cuprizone-induced murine model of demyelination/remyelination. Toxicol Lett. (2007) 169:205–13. 10.1016/j.toxlet.2007.01.010 17317045

[50] Sachs HH Bercury KK Popescu DC Narayanan SP Macklin WB. A new model of cuprizone-mediated demyelination/remyelination. ASN Neuro. (2014) 6:1759091414551955. 10.1177/1759091414551955 25290063 PMC4187018

[51] Bai CB Sun S Roholt A Benson E Edberg D Medicetty S. A mouse model for testing remyelinating therapies. Exp Neurol. (2016) 283:330–40. 10.1016/j.expneurol.2016.06.033 27384502 PMC5207347

[52] Liebetanz D Merkler D. Effects of commissural de- and remyelination on motor skill behaviour in the cuprizone mouse model of multiple sclerosis. Exp Neurol. (2006) 202:217–24. 10.1016/j.expneurol.2006.05.032 16857191

[53] Hibbits N Pannu R Wu TJ Armstrong RC. Cuprizone demyelination of the corpus callosum in mice correlates with altered social interaction and impaired bilateral sensorimotor coordination. ASN Neuro. (2009) 1:e00013. 10.1042/AN20090032 19650767 PMC2784600

[54] Chao CC Gekker G Hu S Sheng WS Shark KB Bu DF. kappa opioid receptors in human microglia downregulate human immunodeficiency virus 1 expression. Proc Nat Acad Sci. (1996) 93:8051–6. 10.1073/pnas.93.15.8051 8755601 PMC38873

[55] Knapp PE Maderspach K Hauser KF. Endogenous opioid system in developing normal and jimpy oligodendrocytes: mu and kappa opioid receptors mediate differential mitogenic and growth responses. Glia. (1998) 22:189–201. 10.1002/(SICI)1098-1136(199802)22:2&lt;189::AID-GLIA10&gt;3.0.CO;2-U 9537839

[56] Belcheva MM Clark AL Haas PD Serna JS Hahn JW Kiss A. Mu and kappa opioid receptors activate ERK/MAPK via different protein kinase C isoforms and secondary messengers in astrocytes. J Biol Chem. (2005) 280:27662–9. 10.1074/jbc.M502593200 15944153 PMC1400585

[57] Chen C Willhouse AH Huang P Ko N Wang Y Xu B. Characterization of a Knock-In Mouse Line Expressing a Fusion Protein of kappa Opioid Receptor Conjugated with tdTomato: 3-Dimensional Brain Imaging via CLARITY. eNeuro. (2020) 7:ENEURO.0028-0020. 10.1523/ENEURO.0028-20.2020 32561573 PMC7385665

[58] Gudi V Gingele S Skripuletz T Stangel M. Glial response during cuprizone-induced de- and remyelination in the CNS: lessons learned. Front Cell Neurosci. (2014) 8:73. 10.3389/fncel.2014.00073 24659953 PMC3952085

