# Supplementary material for: The Salvinorin Analogue, Ethoxymethyl Ether Salvinorin B, Promotes Remyelination in Preclinical Models of Multiple Sclerosis
Source: Front Neurol. 2021 Dec 20;12:782190. doi: 10.3389/fneur.2021.782190 (PMC8721439; doi:10.3389/fneur.2021.782190)
Supplement: Supplementary file 1 [file Data_Sheet_1.PDF]

Spinal cord infiltration quantification – Luxol fast blue

1. Raw image
2. Crop to region of interest from central canal: select area, then image → crop
3. Subtract background: Process → Subtract background
4. Split channels: Image → colour → split channels (select the red channel)

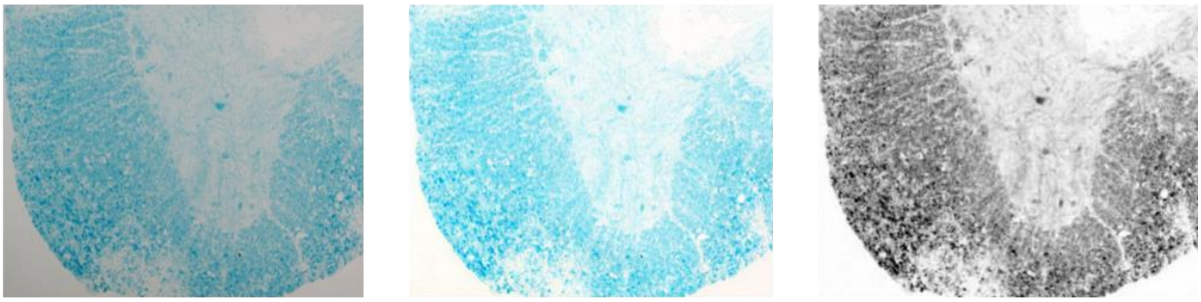

4. Set threshold to include myelinated area and exclude grey matter: Image → Adjust → threshold → set
5. Select white matter area with freehand selections

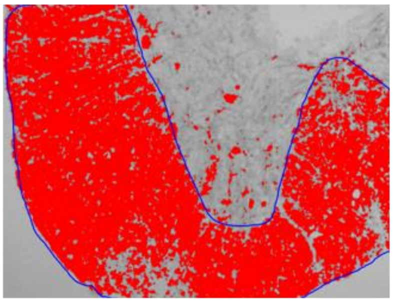

6. Analyse → measure

Supplementary Figure 1. Using ImageJ software, images from luxol fast blue-stained sections were converted to a red, green, blue (RGB) stack, and the red filter was selected to give the highest contrast. The region of interest was selected from the central canal, down the lateral canal, and across the ventral column. Using the selected region, the threshold was set to include white matter and exclude grey matter regions. The white matter area was selected, and the percentage area of myelin stain measured.

Spinal cord infiltration quantification – H&E

1. Raw image
2. Crop to region of interest from central canal: select area, then image → crop
3. Subtract background: Process → Subtract background
4. Split channels: Image → colour → split channels (select the red channel)

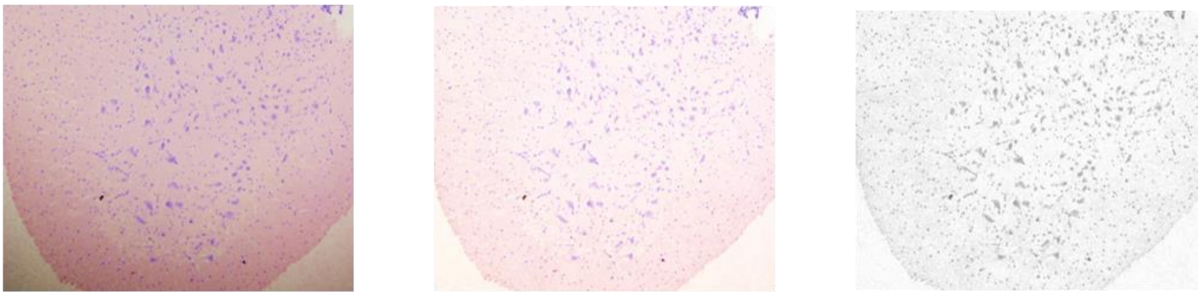

4. Set threshold to include infiltrating nuclei: Image → Adjust → threshold → set
5. Select white matter area with freehand selections

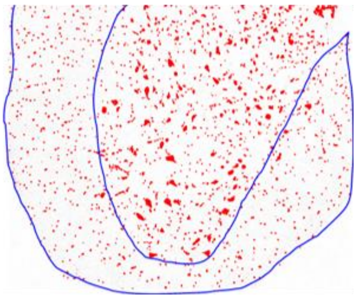

6. Analyse → measure

Supplementary Figure 2. Using ImageJ software, images from hematoxylin and eosin (H&E)-stained sections were converted to a red, green, blue (RGB) stack, and the red filter was selected to give the highest contrast. The region of interest was selected from the central canal, down the lateral canal, and across the ventral column. Using the selected region, the threshold was set to include infiltrating cell nuclei. The white matter area was selected, and the percentage area of infiltration was measured.

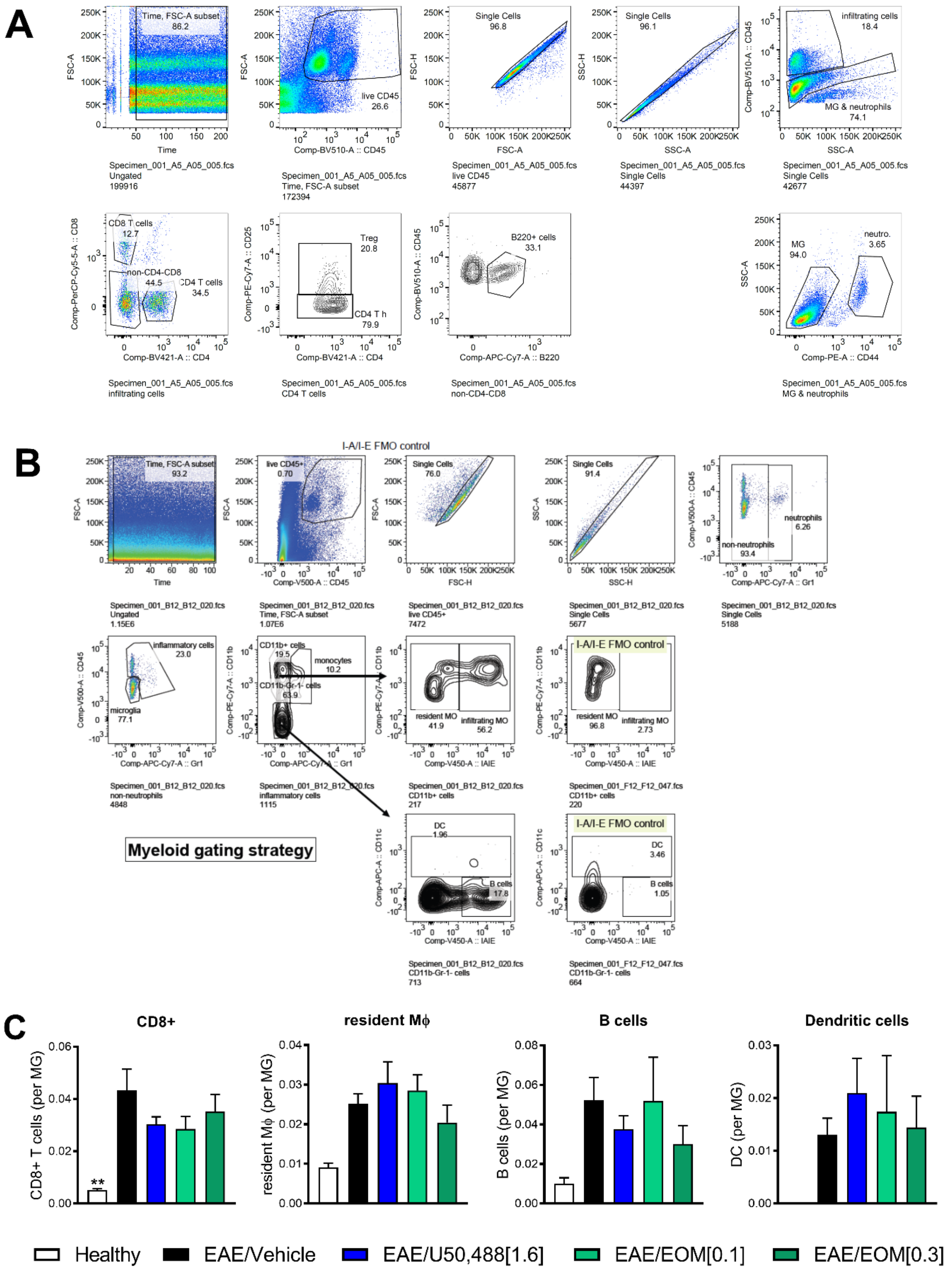

Supplement Figure 3. CNS-infiltrating immune cells after daily EOM SalB or U50,488 treatment in experimental autoimmune encephalomyelitis (EAE) mice. All infiltrating immune cells were identified by CD45^{high} expression and the relative number of cells is expressed as a ratio to microglia. **(A)** Gating strategy for the T cell and B cell panel in the brain. **(B)** Gating strategy for the myeloid cell panel in the brain. **(C)** Analysis of CD8+ T cells, resident

macrophages, B cells and dendritic cell populations from healthy, vehicle, EOM SalB (0.1 or 0.3 mg/kg) and U50,488 (1.6 mg/kg) treated brain tissue. Shown are the results from 3 independent experiments with 8–12 mice per group. Data are mean ± SEM. Ordinary one-way ANOVA for days in recovery and relapse data with Dunnett's multiple comparison test compared to vehicle treatment.

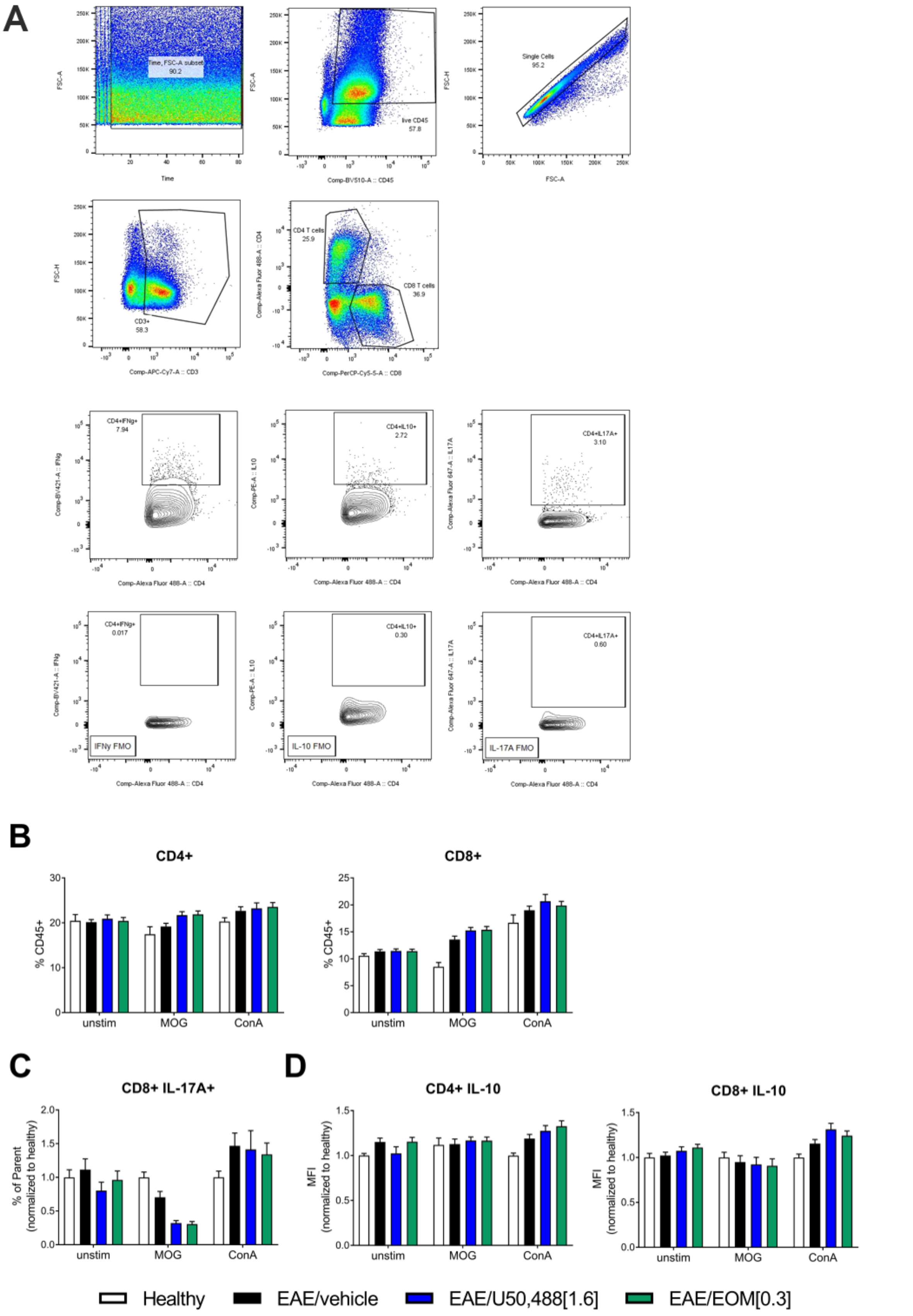

Supplementary Figure 4. (A) Gating strategy for the analysis of splenocyte intracellular cytokines following the experimental autoimmune encephalomyelitis (EAE) model. CD4+ T

cells and CD8+ T cells were defined following the gating strategy above. IFNγ and IL-17A were quantified as the frequency of parent (CD4+ or CD8+), and results normalized to healthy controls. IL-10 was quantified as mean fluorescence intensity (MFI), and results normalized to healthy controls. **(B)** There were no differences in the percentage of CD4+ and CD8+ T cells in response to treatment or stimulation conditions. **(C)** CD8+ T cell IL-17A (as frequency of parent, CD8+) from splenocytes of EAE animals treated daily from onset with vehicle, U50,488 (1.6 mg/kg), or EOM SalB (0.3 mg/kg). **(D)** CD4+ and CD8+ T cell IL-10 MFI (as frequency of parent, CD4+ or CD8+) from splenocytes of EAE animals treated daily from onset with vehicle, U50,488 (1.6 mg/kg), or EOM SalB (0.3 mg/kg).

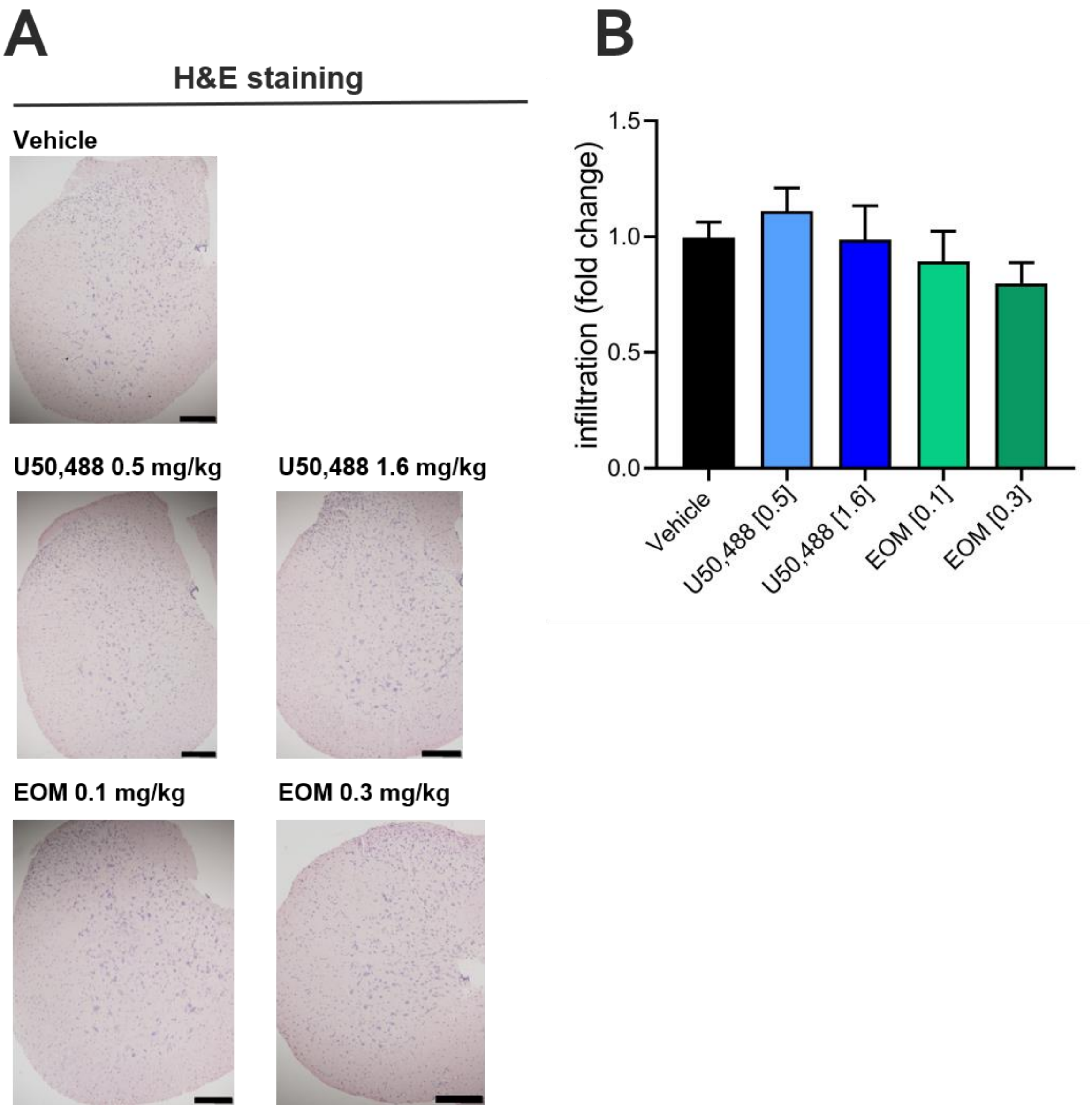

Supplementary Figure 5. (A) Paraffin-embedded spinal cord sections assessed by hematoxylin and eosin (H&E) staining. Representative images of spinal cord sections from experimental autoimmune encephalomyelitis (EAE) animals treated with vehicle, U50,488 (0.5 mg/kg, 1.6 mg/kg) or EOM SalB (0.1 mg/kg, 0.3 mg/kg). Results are shown from 3 independent EAE experiments, n=4-9 mice per group. Two sections from the cervical spinal cord averaged per animal. Scale bars, 100 µm. **(B)** Quantification of leukocyte infiltration as fold change from vehicle. Kruskal-Wallis with Dunn's multiple comparison test. Data presented as mean ± SEM.
